# Halophyte‐Derived *Kushneria* Strains Enhance Salt Tolerance and Rhizosphere Dynamics in Cabbage

**DOI:** 10.1111/pce.70234

**Published:** 2025-10-12

**Authors:** Yuxin Peng, Ju Huck Lee, Cha Young Kim, Jiyoung Lee

**Affiliations:** ^1^ Korean Collection for Type Cultures (KCTC), Biological Resource Center Korea Research Institute of Bioscience and Biotechnology Jeongeup South Korea; ^2^ KRIBB School of Biotechnology University of Science and Technology (UST) Daejeon South Korea

**Keywords:** biofilm formation, halotolerant bacteria, plant growth‐promoting bacteria, rhizosphere microbiome, salt stress

## Abstract

Halophytic plants harbour salt‐tolerant bacteria that enhance resilience to salinity. In this study, two highly halotolerant *Kushneria* isolates, *K. konosiri* (Kk) and *K. marisflavi* (Km), were obtained from the halophyte *Suaeda maritima*. Both strains tolerated up to 25% NaCl and promoted *Arabidopsis thaliana* growth under salt stress by producing indole‐3‐acetic acid, proline, and extracellular polysaccharides that mitigated osmotic stress. Inoculation with Kk or Km increased shoot and root biomass and reduced intracellular Na⁺ and reactive oxygen species. Their agricultural potential was tested in cabbage (*Brassica rapa*), where both isolates alleviated salinity‐induced growth inhibition. A combined inoculum (Kkm) showed enhanced efficacy, significantly increasing shoot biomass (1.26‐fold vs. Kk; 1.23‐fold vs. Km) and dry weight (1.19‐fold vs. Kk; 1.13‐fold vs. Km). Kkm treatment also improved the K⁺/Na⁺ ratio and proline accumulation. Microbial profiling revealed that Kkm enriched *Bacillus* species in the rhizosphere and promoted greater biofilm formation than single strains. These findings demonstrate that *Kushneria* isolates function as salt‐tolerant plant growth‐promoting bacteria, enhancing ion homoeostasis, stress protection, and rhizosphere restructuring. This study highlights the potential of halophyte‐derived microbial consortia to improve crop salt tolerance in agriculture.

## Summary statement

Halophyte‐derived *Kushneria* strains were evaluated for their ability to enhance salt tolerance and shape rhizosphere microbial dynamics in cabbage. This study demonstrates the potential of these endophytic bacteria as plant growth‐promoting inoculants, offering a sustainable strategy to mitigate salt stress in crops. Our findings provide new insights into the role of halophyte‐associated microbiomes in crop improvement under challenging environments.

## 1 Introduction

Salinity stress disrupts water uptake, nutrient absorption and overall physiological functions in plants, leading to stunted growth, chlorosis, and reduced plant quality and yield. It notably diminishes crop biomass and chlorophyll content, thereby undermining photosynthetic efficiency (Parida and Das 2005). Additionally, salinity stress induces the accumulation of reactive oxygen species (ROS) within plant cells, exacerbating oxidative damage and further intensifying stress symptoms (Gill and Tuteja 2010). The primary sites of ROS production under salinity stress are the electron transport chains in chloroplasts and mitochondria (Mittler 2002). Elevated salt ions concentration in plant tissues can reach toxic levels, further compounding the harmful effects of stress (Zhu 2001). These physiological disturbances underscore the urgent need for effective strategies to mitigate the impacts of salinity stress on plant growth and survival.

With the increasing salinization of agricultural lands, developing strategies to enhance crop salt tolerance has become critical (Ammari et al. 2015). A promising area of research involves the use of halophytes, salt‐tolerant plants with unique physiological and biochemical adaptations that enable them to thrive in high‐salinity environments. Halophytes provide valuable insights into plant salt tolerance mechanisms, and previous studies have shown that their salt tolerance is closely linked to their rhizosphere microbial communities (Qin et al. 2018). These plants harbour unique bacterial communities capable of conferring salt tolerance to non‐halophytic, or glycophytic plants. Such microbes adapt to high‐salinity environments and assist host plants in coping with salt stress through various mechanisms (Dragojevic et al. 2023). Recent studies have highlighted the potential of halophyte‐derived bacteria to promote plant growth under saline conditions. For instance, co‐inoculation of maize varieties with co‐cultures of *Bacillus* sp. and *Arthrobacter pascens* isolated from *Atriplex leucoclada* and *Suaeda fruticosa* has been shown to increase osmotic solutes accumulation, including sugars and proline, and enhance the activities of antioxidant enzymes, such as superoxide dismutase (SOD), peroxidase (POD), catalase (CAT), and ascorbate peroxidase (APX) (Ullah and Bano 2015). Additionally, *Pseudomonas* spp. isolated from *Salicornia europaea* produce indole‐3‐acetic acid (IAA), a phytohormone that stimulates root growth and improves nutrient absorption in saline environments (Sridevi et al. 2022).

While several studies have highlighted the potential of halophyte‐associated bacteria to enhance salt tolerance in plants, their application to conventional glycophyte crops remains limited. The mechanisms by which these bacteria influence the rhizosphere microbial communities of glycophytes and induce biofilm formation in beneficial strains, thereby enhancing salt tolerance, are not yet fully understood. Biofilms, complex structures formed by microbial communities secreting extracellular polymeric substances (EPS), create protective microenvironments that shield microbes from environmental stresses and facilitate intercellular communication and resource sharing (Xiong et al. 2020; Yin et al. 2019). These microenvironments significantly enhance microbial survival and functionality, enabling them to perform effectively under adverse conditions (Ikuma et al. 2013). Moreover, biofilms promote synergistic interactions among microbial species, supporting complementary physiological functions that increase the adaptability of the microbial community to environmental changes (Yin et al. 2019). Such synergy is crucial in helping plants cope with salt stress. Additionally, biofilms strengthen plant‐microbe interactions, fostering stable symbiotic relationships that enhance plant growth and resilience (Banerjee et al. 2019; Gogoi et al. 2021).

The halophilic bacterial genus *Kushneria* has garnered significant attention due to its unique adaptations to high‐salinity environments and promising agricultural applications (Meinzer et al. 2023). Isolated from extreme saline habitats such as salt marshes, saline soils, and hypersaline lakes, *Kushneria* species are valuable models for understanding salt tolerance mechanisms and developing microbial strategies to mitigate salt stress in crops (Panwar et al. 2016). In plant‐microbe interactions, *Kushneria* isolates function effectively as plant growth‐promoting rhizobacteria (PGPR), demonstrating beneficial effects in various host plants (Szymańska et al. 2022; Zhu et al. 2011). These strains enhance plant growth under saline conditions by improving nutrient uptake, producing phytohormones such as IAA, and solubilizing essential minerals like phosphorus (Yuan et al. 2021; Zhu et al. 2011). In addition, *Kushneria* inoculation enhances plant antioxidant capacity, thereby alleviating oxidative stress under salinity (Bekkaye et al. 2023; Kim and Sang 2023).

Although previous studies have reported the plant growth‐promoting potential of halophilic bacteria such as *Kushneria* or *Bacillus* individually, the synergistic interactions between these genera, particularly in the context of biofilm formation under saline conditions, remain largely unexplored. This study is the first to demonstrate that co‐inoculation of *Kushneria* isolates can enhance *Bacillus*‐associated biofilm development, suggesting a novel microbial consortium‐based strategy to improve plant performance in saline soils.

## 2 Results

### 2.1 Isolation and Characterisation of Salt‐Tolerant Endophytic Bacteria From Halophytes

**Figure 1:**
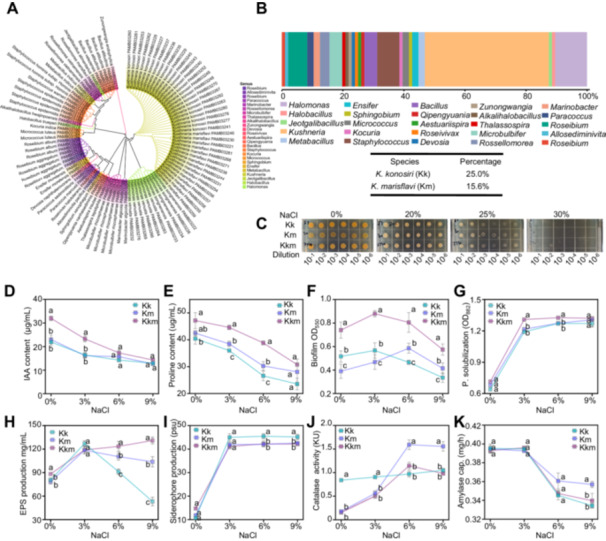
Isolation and physiological characterisation of salt‐tolerant *Kushneria* isolates. (A) Phylogenetic analysis of isolated bacterial strains, highlighting distinct genera including *K. konosiri* (Kk) and *K. marisflavi* (Km), which represent 25.0% and 15.6% of the endophytic bacterial community, respectively. (B) Relative abundance of identified endophytic bacterial species. (C) Growth of *Kushneria* isolates on TSA medium supplemented with increasing NaCl concentrations, showing salt tolerance up to 30%. (D–K) Physiological traits of the isolates, (D) IAA production, (E) proline accumulation, (F) biofilm formation, (G) phosphate solubilisation, (H) EPS production, (I) siderophore production, (J) catalase activity, and (K) amylase activity. All experiments were conducted in triplicate with consistent results. Statistical analysis was performed using one‐way ANOVA; different letters indicate significant differences (*p* < 0.05).

In this study, *Suaeda maritima* plants were collected from the coastal reclaimed land of Saemangeum, Gimje‐si, Jeollabuk‐do, Republic of Korea (35°50'21.012″ N, 126°35'12.588″ E). A total of 96 bacterial strains were isolated and identified from these plants. NCBI BLAST analysis revealed that the genus *Kushneria* was the most abundant, with *K. konosiri* (Kk) and *K. marisflavi* (Km) as the predominant species, accounting for 25.0% and 15.6% of the bacterial population, respectively (Figure 1A,B). Salt tolerance assays demonstrated that both strains could tolerate NaCl concentrations up to 25% (w/v), while a bacterial consortium consisting of two *Kushneria* species (Kkm) showed no significant improvement in salt tolerance at 20%–25% NaCl (w/v) (Figure 1C).

### 2.2 Biochemical Properties and Salt Tolerance of *Kushneria* Isolates

To investigate the biochemical traits associated with plant growth effect of *Kushneria* isolates, we evaluated the ability of the bacteria to produce various growth‐promoting compounds, including IAA, proline, EPS, biofilms, and siderophores at different concentrations of NaCl.

With increasing salinity, IAA and proline production significantly decreased in individual strains, but the Kkm consortium effectively delayed the salinity‐induced reduction in IAA and proline production (Figure 1D,E). All treatments (Kk, Km and Kkm) and salinity levels (0%, 3%, 6% and 9% w/v NaCl), the Kkm treatment maintained the highest proline content, indicating an enhanced ability to withstand osmotic stress under saline conditions (Figure 1E). Biofilm formation was also significantly enhanced in Kkm treatments, particularly under higher NaCl concentrations (Figure 1F). Phosphorus solubilisation increased from 0% to 3% NaCl and then stabilised from 3% to 9% NaCl, with Kkm showing slightly higher solubilisation (Figure 1G). EPS production peaked at 3% NaCl for all groups, with Kkm maintaining high EPS levels even at 9% NaCl, whereas Kk and Km showed a decline (Figure 1H). Siderophore production also increased with higher salt stress in all groups, with Kkm exhibiting the most significant enhancement (Figure 1I). Catalase and amylase activities also varied with salinity, showing distinct activity patterns in Kkm compared to the individual strains (Figure 1J,K). Growth curve analysis further supported these observations, as Kkm consortium exhibited improved growth under 3% and 6% NaCl compared to individual strains, suggesting a synergistic effect under moderate salt stress. However, this effect was not observed under nonsaline conditions (0%) or high‐salinity (≥ 9%) conditions, indicating that the synergistic interaction is specific to intermediate salt concentrations (Supporting Information S1: Figure S1).

### 2.3 Enhancement of *Arabidopsis* Growth Under Saline Conditions by *Kushneria* Isolates

**Figure 2:**
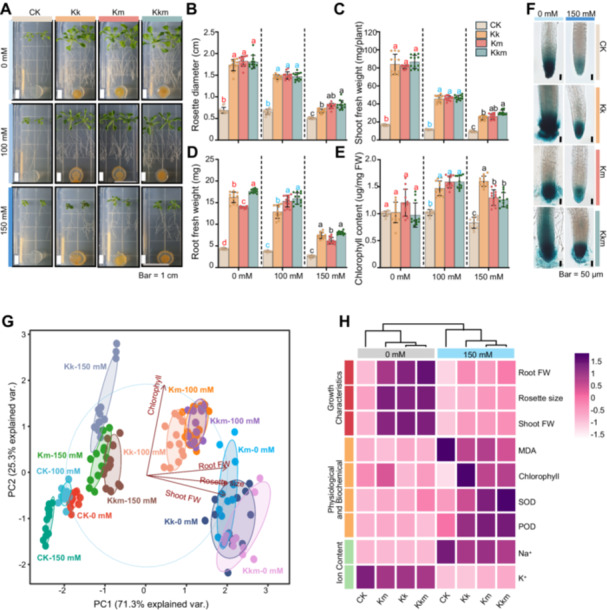
Growth‐promoting effects of *Kushneria* isolates inoculation on *Arabidopsis* seedlings under saline conditions. (A) Representative image of *Arabidopsis* seedlings inoculated with *Kushneria* isolates, showing plant growth under salt stress. Scale bar = 1 cm. (B) Rosette diameter of seedling. (C) Shoot fresh weight, representing above‐ground biomass. (D) Root fresh weight, representing below‐ground biomass. (E) Chlorophyll content of seedlings. FW, fresh weight. Red, blue, and black letters indicate statically significant differences under 0,100, and 150 mM NaCl treatment, respectively (two‐way ANOVA, *p* < 0.05). (F) GUS staining of DR5:GUS transgenic root tips under 0 mM and 150 mM NaCl, visualising auxin distribution. Scale bar = 50 μM. (G) Principal component analysis (PCA) of growth parameters. (H) Heatmap and cluster analysis illustrating similarities and differences among treatment groups. [Color figure can be viewed at wileyonlinelibrary.com]

We evaluated the effects of *Kushneria* isolates on *Arabidopsis* seedling growth using 1/2 MS medium supplemented with 0, 100 and 150 mM NaCl. Both *K. konosiri* (Kk) and *K. marisflavi* (Km) significantly improved plant tolerance to salt stress, enhancing rosette diameter, shoot and root fresh weights, and chlorophyll content. Under 100 mM NaCl, rosette diameters for Kk, Km, and Kkm treatments reached 1.45, 1.49 and 1.47 cm, respectively, compared to 0.65 cm in the untreated control (CK). At 150 mM NaCl, rosette diameters were 0.67 cm (Kk), 0.79 cm (Km), and 0.82 cm (Kkm), while the control showed a significantly smaller diameter of 0.51 cm (Figure 2A,B).

Under nonsaline conditions (0 mM NaCl), shoot fresh weight for Kk, Km, and Kkm treated *Arabidopsis* seedlings were 84.08, 81.05, and 86.65 mg, respectively, compared to 16.60 mg in CK. At 100 mM NaCl, shoot fresh weights decreased but remained significantly higher than CK, reaching 45.59 mg (Kk), 46.18 mg (Km) and 46.92 mg (Kkm), while CK plants showed only 11.53 mg. Under 150 mM NaCl, shoot weights were 24.48 mg (Kk), 26.15 mg (Km), and 30.45 mg (Kkm), compared to 9.33 mg in CK (Figure 2C).

Similarly, root fresh weight was highest in Kkm‐treated plants (17.65 mg) under 0 mM NaCl, followed by Kk (16.23 mg), Km (13.90 mg), and CK (4.31 mg). Under 100 mM NaCl, values remained elevated in treated groups, 12.89 mg (Kk), 15.34 mg (Km), and 15.95 mg (Kkm) compared to 3.73 mg in CK. At 150 mM NaCl, root fresh weights declined but still exceed CK values, measuring 7.40 mg (Kk), 6.19 mg (Km), and 8.01 mg (Kkm), while 2.57 mg in CK (Figure 2D).

Chlorophyll content showed no significant differences among treatments under nonsaline conditions. However, at 100 mM NaCl, chlorophyll increased in all treated plants, with 1.47 μg/mg (Kk) and 1.59 μg/mg (Km and Kkm), compared to 1.02 μg/mg in CK. At 150 mM NaCl, the highest content was observed in Kk‐treated plants (1.60 μg/mg), followed by Km (1.30 μg/mg) and Kkm (1.26 μg/mg), while CK remained lower at 0.83 μg/mg fresh weight (Figure 2E). Statistical analysis by two‐way ANOVA showed that salt stress and bacterial inoculation had significant effects on all measured traits, including rosette diameter, shoot and root fresh weights, and chlorophyll content (Supporting Information S1: Table S2).

To determine whether improved root development was associated with auxin accumulation, *Arabidopsis* DR5::GUS reporter lines were used to visualise IAA distribution (Naser and Shani 2016). Under salt stress, CK plants showed reduced IAA in root tips, while Kk‐, Km‐, and particularly Kkm‐inoculated seedlings displayed enhanced IAA accumulation, with the strongest GUS signal in the Kkm group (Figure 2F).

Principal component analysis (PCA) revealed clear separation between control and *Kushneria*‐inoculated groups. PC1, accounting for 71.3% of total variance, was mainly driven by biomass‐related traits such as rosette diameter, shoot and root fresh weights (Figure 2G). Inoculated plants clustered positively along PC1, reflecting enhanced growth, whereas salt‐stressed controls were negatively positioned, indicating reduced biomass and elevated stress. Hierarchical clustering heatmaps (Figure 2H) supported these findings. Treated plants (Kk, Km, Kkm) maintained higher chlorophyll content and favourable ion balance (increased K^+^, decreased Na^+^) relative to CK under the salt stress. Furthermore, antioxidant enzymes activities (SOD and POD) were elevated in inoculated plants, while malondialdehyde (MDA) accumulation was markedly higher in CK, indicating greater lipid peroxidation, and oxidative damage.

Collectively, these physiological and biochemical enhancement demonstrate that *Kushneria* inoculates promote salt stress tolerance in *Arabidopsis* through coordinated mechanisms involving hormonal regulation, redox balance, and improved ion homoeostasis.

### 2.4 Reduction of Na^+^ and ROS Accumulation in *Arabidopsis* Seedlings by *Kushneria* Isolates

**Figure 3:**
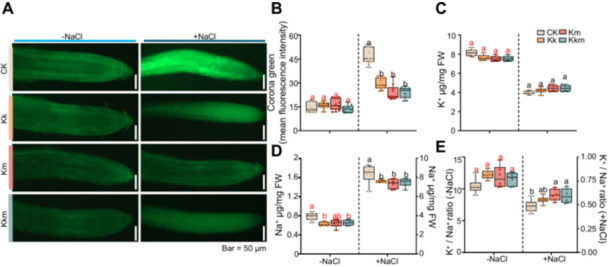
Effects of *Kushneria* isolates inoculation on Na⁺ uptake in *Arabidopsis* seedlings under salt stress. (A) CoroNa Green staining of root tips to visualise Na⁺ uptake. Scale bar = 50 μM. (B) Quantification of CoroNa Green fluorescence intensity 24 h after inoculation with *Kushneria* strains under control (0 mM NaCl) and salt stress (200 mM NaCl) conditions. (C) K⁺ accumulation, (D) Na⁺ accumulation, and (E) K⁺/Na⁺ ratio in *Arabidopsis* seedlings measured 7 days after inoculation with *Kushneria* strains, under both nonsaline and saline (150 mM NaCl) conditions. Red and black letters indicate significant differences under nonsaline and saline conditions, respectively (two‐way ANOVA, *p* < 0.05). [Color figure can be viewed at wileyonlinelibrary.com]

To determine whether *Kushneria* isolates could alleviate sodium accumulation in salt‐stressed *Arabidopsis* seedling, CoroNa Green, a Na^+^‐selective fluorescent dye was employed. Under salinity conditions, root tips of untreated seedlings displayed strong fluorescence signals, indicative of excessive Na^+^ accumulation. In contrast, seedlings treated with Kk, Km or the Kkm exhibited substantially reduced fluorescence intensities, closely resembling non‐stressed controls (Figure 3A,B). Quantitative analysis confirmed a significantly lower in Na⁺ content and improved K⁺/Na⁺ ratio in all *Kushneria*‐treated groups, indicating effective alleviation of ionic stress (Figure 3C–E).

**Figure 4:**
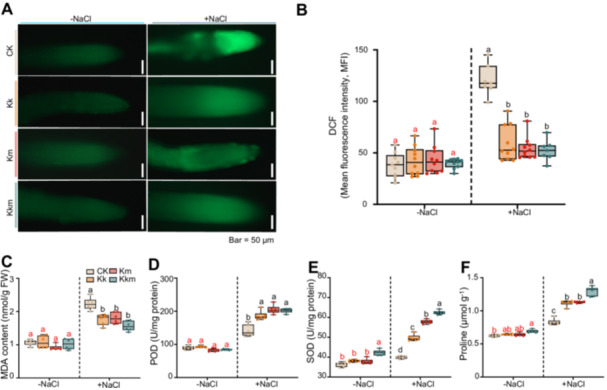
Effects of *Kushneria* isolates inoculation on antioxidant activity and ROS levels in *Arabidopsis* seedlings under saline conditions. (A) Reactive oxygen species (ROS) levels in roots visualised by DCFH‐DA fluorescence 24 h after inoculation with *Kushneria* strains under control (0 mM NaCl) and saline (200 mM NaCl) conditions. Scale bar = 50 μM. (B) Quantification of DCF fluorescence intensity indicating ROS accumulation. (C) Malondialdehyde (MDA) content as an indicator of lipid peroxidation and oxidative damage. (D) Peroxidase (POD) activity as a measure of antioxidant response. (E) Superoxide dismutase (SOD) activity, showing enzymatic defence against oxidative stress. Antioxidant activities were measured 7 days postinoculation under both nonsaline and saine (150 mM NaCl) conditions. (F) Proline content in *Arabidopsis* seedlings under nonsaline and saline conditions. Red and black letters indicate significant differences under nonsaline and saline conditions, respectively (two‐way ANOVA, *p* < 0.05). [Color figure can be viewed at wileyonlinelibrary.com]

To evaluate oxidative stress, reactive oxygen species (ROS) levels in root tissues were assessed using DCFH‐DA staining. All three treatments significantly reduced ROS accumulation, accompanied by notable decreases in malondialdehyde (MDA) content, a marker of lipid peroxidation (Figure 4A–C). Furthermore, antioxidant enzyme activities, including peroxidase (POD) and superoxide dismutase (SOD), were significantly elevated in inoculated seedlings, with the highest activity observed in the Kkm treatment (Figure 4D,E).

Proline levels, indicative of osmotic adjustment capacity, were significantly incisolates inoculation on cabbage growth in salinereased under salt stress in *Kushneria* treated seedlings, with Kkm‐treated plants exhibiting the greatest accumulation (Figure 4F). The two‐way ANOVA results indicate that the pronounced interaction effects observed in several key physiological traits indicate that the positive impact of bacterial treatment is significantly enhanced under salt stress, supporting their potential use as effective salt‐tolerant plant growth‐promoting bacteria (St‐PGPB) for improving crop performance in saline agricultural environments (Supporting Information S1: Table S2).

### 2.5 Modulation of Salt Stress‐Responsive Gene Expression by *Kushneria* Strains

To investigate the influence of *Kushneria* strains on salt‐responsive gene expression, we performed qRT‐PCR analysis of three key genes, *RD29A*, *RD20*, and *KIN1* in *Arabidopsis* seedlings exposed to 0, 100 and 150 mM NaCl for 3, 6 and 12 h. In untreated control plants, all genes were rapidly induced by salt stress, with expression peaking at 3 h. Among them, *RD20* showed the highest induction, particularly at 150 mM NaCl (Supporting Information S1: Figure S2). In contrast, *Kushneria*‐inoculated seedlings exhibited attenuated and delayed transcriptional responses. Gene expression typically peaked at 6 h, suggesting a moderated stress perception. In the Kkm‐treated group, both *RD29A* and *RD20* showed significantly reduced expression levels at both 100 and 150 mM NaCl, particularly at 6 h. By 12 h, differences in gene expression between treated and untreated plants were largely diminished. *KIN1* expression in the Kkm group remained comparable to that in Kk‐ and Km‐ treated seedlings, showing no further repression.

These results suggest that *Kushneria*, particularly the Kkm consortium, modulates plant response to salt stress by delaying and dampening the transcriptional activation of key stress‐related gene. Although phenotypic improvements were comparable to those of single‐strain treatments, the distinct regulatory pattern points to a potential synergistic mechanism at the molecular level.

### 2.6 Alleviation of Salt Stress in Soil‐Grown Cabbage Through *Kushneria* Isolates Inoculation

Although the Kkm consortium did not exhibit significantly better performance than the individual strains Kk and Km in the *Arabidopsis* growth assay on 1/2 MS agar (Figure 2), it exhibited distinct biochemical traits that suggest a potential advantage in more complex environments (Figure 1). In particular, Kkm consortium significantly increased biofilm formation under nonsaline (0% NaCl), independent of cell density (Supporting Information S1: Figure S3A,B) a trait critical for effective root colonisation. These findings imply that Kkm consortium may be better suited for soil‐based system, where biofilm‐mediated interaction play a key role in microbial persistence and plant growth promotion.

#### 2.6.1 Plant Growth Performance Under Salt Stress

**Figure 5:**
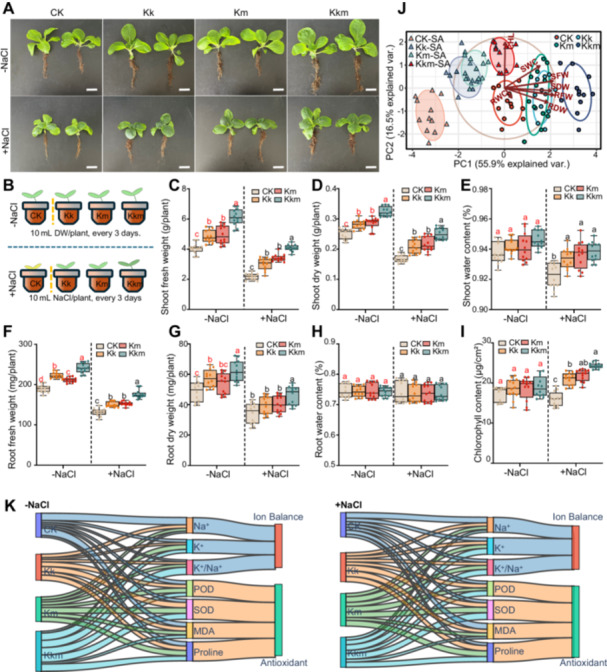
Effects of *Kushneria* isolates inoculation on cabbage growth in saline soil. (A) Representative images of cabbage plants under nonsaline (DW) and saline (200 mM NaCl, SA) conditions. (B) Experimental design showing inoculation and irrigation setup. (C–E) Shoot traits: (C) fresh weight, (D) dry weight, and (E) water content. (F–H) Root traits: (F) fresh weight, (G) root dry weight, and (H) root water content. (I) Chlorophyll content in cabbage leaves. FW, fresh weight. Scale bar = 4 cm. Red and black letters indicate significant differences under nonsaline and saline conditions, respectively (two‐way ANOVA, *p* < 0.05). (J) Principal component analysis (PCA) of growth parameters. (K) Sankey diagram illustrates treatment effects on ion balance (Na⁺, K⁺, K⁺/Na⁺) and antioxidant responses (POD, SOD, MDA, and proline) under nonsaline (−NaCl) and saline (+NaCl) conditions. Flow widths represent normalised contributions (min‐scaling with offset). [Color figure can be viewed at wileyonlinelibrary.com]

To assess the agronomic potential of *Kushneria* isolates in enhancing salt stress tolerance, we conducted growth experiments using cabbage (*Brassica rapa*) under both nonsaline and saline conditions. Under nonsaline conditions, all inoculated groups showed increased biomass compared to control (CK), with Kk, Km yielding 4.84, 5.94 and 6.12 g/plant, respectively, compared to 4.05 g/plant in CK. Under salt stress, although overall biomass decreased, Kkm‐treated plants retained the highest shoot weight (4.08 g/plant), followed by Km (3.42 g/plant) and Kk (3.02 g/plant), and CK (2.18 g/plant) (Figure 5A,C). Similarly, Kkm‐inoculated plants maintained the highest root fresh weight under both nonsaline (240.67 mg/plant) and saline (176.25 mg/plant) conditions. Kk and Km also improved root biomass relative to CK (Figure 5F). Kkm inoculation resulted in the highest shoot dry weight (0.32 g/plant) under nonsaline conditions and maintained better dry biomass (0.25 g/plant) than the CK (0.17 g/plant). Root dry weight followed similar trends (Figure 5D,G). Shoot water content remained comparable among all treatments in nonsaline conditions but was significantly higher in inoculated plants under salt stress. Kkm maintained the highest shoot water content (93.83%) compared to CK (92.23%), though root water content was not significantly affected (Figure 5E,H).

Chlorophyll content measurements provided additional evidence of the growth‐promoting effects of *Kushneria* treatments. Among all treatments, plants inoculated with the Kkm consortium exhibited the highest chlorophyll levels, reaching 18.98 μg/cm² under nonsaline conditions and 24.29 μg/cm² under saline conditions. Plants treated with Kk and Km also showed elevated chlorophyll contents compared to the control (CK), with values of 18.57 and 18.68 μg/cm² under nonsaline conditions, and 20.94 and 21.93 μg/cm² under salt stress, respectively. In contrast, control plants exhibited the lowest chlorophyll contents, measuring 17.37 μg/cm² without salt and 15.95 μg/cm² under salinity (Figure 5I). These findings demonstrate a clear gradient of effectiveness among treatments, with the Kkm consortium producing the most significant improvements. This consistent enhancement suggests a synergistic effect between the two *Kushneria* strains, leading to improved chlorophyll retention, increased biomass accumulation, and greater photosynthetic efficiency under saline stress.

Principal component analysis (PCA) revealed that PC1 accounted for 55.9% of total variation. Kkm‐treated plants clustered positively with biomass and chlorophyll‐associated traits, while salt‐stressed CK plants grouped negatively, aligning with Na⁺ content and reduced water status (Figure 5J). These findings suggest that Kkm has a distinct and synergistic effect on plant performance under saline conditions. Two‐way ANOVA further confirmed that both salt stress and bacterial inoculation significantly influenced growth and physiological traits (Supporting Information S1: Table S2).

#### 2.6.2 Ion Balance and Antioxidant Enzyme Activities

Under salt stress, CK plants showed high Na⁺ content (26.24 mg/g DW) and a low K⁺/Na⁺ ratio (0.88). In contrast, Kkm‐treated plants had the lowest Na⁺ levels (20.53 mg/g DW) and the highest K⁺/Na⁺ ratio (1.49), indicating improved ion regulation (Figure 5K, Supporting Information S1: Table S3). Under nonsaline conditions, all treatments slightly elevated SOD, POD and proline levels. Under salinity, Kkm again showed the strongest defence response with the highest SOD (661.00 U/mg protein), POD (70.40 U/mg protein), and lowest MDA (26.50 nmol/g FW), indicating reduced lipid peroxidation. Proline accumulation was also highest in Kkm‐treated plants (3.09 µmol/g FW), followed by Kk (2.93) and Km (2.89), all surpassing CK (2.56) (Figure 5K, Supporting Information S1: Table S3). Two‐way ANOVA confirmed that both salt stress and bacterial inoculation significantly influenced ion content, antioxidant enzyme activities, MDA, and proline levels (Supporting information Table S2).

### 2.7 Rhizosphere Microbial Community Dynamics Under Salt Stress With *Kushneria* Isolates Inoculation

**Figure 6:**
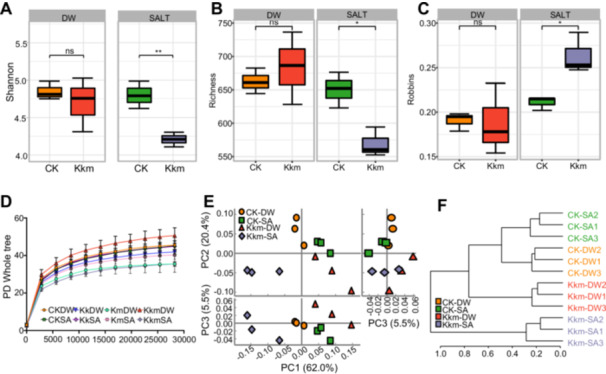
Diversity analysis of rhizosphere microbial communities influenced by *Kushneria* isolates inoculation under saline conditions. (A–C) Alpha diversity metrics, including (A) Shannon diversity, (B) species richness, and (C) Robbins indices, illustrating the effects of *Kushneria* isolates (Kk, Km, consortium Kkm) on microbial community diversity in the rhizosphere under nonsaline (DW) and saline (SA) conditions. (D) Rarefaction curves of phylogenetic diversity (PD). (E) Principal coordinate analysis (PCoA) based on beta diversity. (F) Hierarchical clustering dendrogram using the Jaccard index, visualising similarities and differences among microbial communities across treatment groups. [Color figure can be viewed at wileyonlinelibrary.com]

To determine whether *Kushneria* isolates influence rhizosphere microbial communities, we analyzed the microbiome composition of cabbage rhizospheres under both nonsaline (DW) and saline (SA) conditions. Microbial diversity was assessed using alpha and beta diversity metrics (Figure 6 and Supporting Information S1: Figure S4). Alpha diversity, measured by Shannon index, species richness, and Robbins index, revealed no significant differences between the control (CK) and plants treated with Kk or Km under either condition (Supporting Information S1: Figure S4A–C), suggesting that single‐strain treatments had minimal impact on overall microbial diversity. In contrast, the Kkm consortium under saline conditions (Kkm‐SA) led to a significant reduction in Shannon diversity and species richness, along with an increase in the Robbins index (Figure 6A–C, Supporting Information S1: Figure S4A–C). These changes suggest a shift toward a more specialised microbial community structure. Rarefaction analysis confirmed that phylogenetic diversity (PD) reached saturation with increasing sequence depth, supporting the robustness of the observed trends (Figure 6D).

Beta diversity analysis using principal coordinate analysis (PCoA) further demonstrated a clear divergence in community composition. The Kkm‐SA samples clustered distinctly from all other treatments, indicating a unique microbial community structure under saline conditions (Figure 6E, Supporting Information S1: Figure S4D). These findings demonstrate that Kkm treatment under salt stress distinctly reshapes the rhizosphere microbiome, potentially contributing to enhanced plant resilience in saline environments. This suggests an active role of Kkm in restructuring microbial communities to support plant stress tolerance.

Hierarchical clustering using the Jaccard method also supported these findings. While Kk and Km treatments were not clearly distinguishable from CK, Kkm‐treated samples, particularly under salt stress, formed independent clusters (Supporting Information S1: Figure S4E). When Kk and Km samples were excluded, clearer separation emerged: CK‐DW, CK‐SA, Kkm‐DW, and Kkm‐SA each formed distinct groups, with Kkm‐SA forming a strongly separated cluster (Figure 6F). These results indicate that Kkm inoculation under salt stress distinctly restructures the rhizosphere microbiome, potentially enhancing plant resilience. Unlike single‐strain treatments, the Kkm consortium actively modulates microbial community composition, suggesting its role in engineering a beneficial rhizosphere environment that supports plant adaptation to saline conditions.

### 2.8 Taxonomic Composition and Structural Shifts in Rhizosphere Microbial Communities With *Kushneria* Isolates Inoculation

To explore how *Kushneria* inoculation influences rhizosphere microbiome composition under salt stress, we analyzed taxonomic profiles in cabbage rhizospheres across treatments using Venn diagrams, diversity indices, and taxonomic distribution plots.

**Figure 7:**
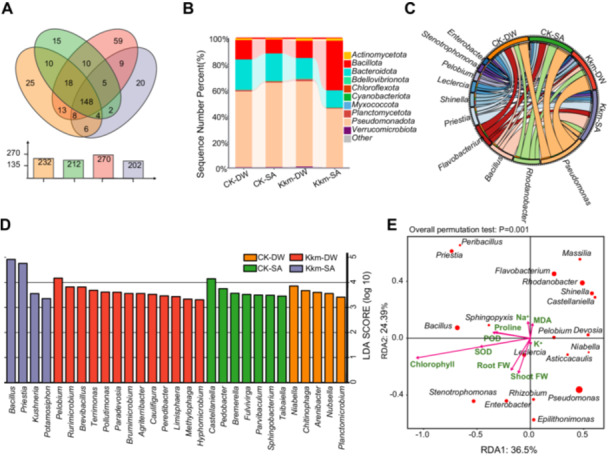
Structural composition of rhizosphere microbial communities under salt stress with *Kushneria* isolates inoculation. (A) Venn diagram shows unique and shared bacterial genera across different treatments. (B) Stacked bar chart illustrating the relative abundance of bacterial phyla in control (CK) and *Kushneria* consortium (Kkm). (C) Chord diagram representing the distribution of dominant genera among the treatments. (D) Linear discriminant analysis (LDA) identifying bacterial genera most significantly impacted by each treatment. (E) Redundancy analysis (RDA) plot displaying correlations between microbial genera and plant traits, with *Bacillus* positively associated with root length, chlorophyll content, and root biomass under Kkm‐SA. [Color figure can be viewed at wileyonlinelibrary.com]

A Venn diagram (Figure 7A) revealed both shared and unique bacterial genera among treatment groups. A total of 148 genera were common across all groups, while CK‐DW, CK‐SA, Kkm‐DW and Kkm‐SA harboured 25, 15, 59 and 20 unique genera, respectively. Notably, Kkm‐DW exhibited the highest total number of genera (270), while Kkm‐SA showed the lowest (202), indicating that both salinity and inoculation substantially altered microbial richness.

Relative abundance analyses at the phylum level (Figure 7B) demonstrated distinct shifts in microbial composition between inoculated and control groups (CK). In particular, Kkm‐SA showed a marked increase in the abundance of *Bacillota*, which includes the genus *Bacillus* (Figure 7C). This enrichment was notably higher than in CK‐SA, suggesting that co‐inoculation with *K. konosiri* and *K. marisflavi* under salt stress promotes beneficial bacterial groups more effectively than either factor alone. Kkm‐SA also differed substantially from CK‐DW, emphasising the interactive effects of salt stress and microbial inoculation.

As shown in Supporting Information S1: Figure S5, the relative abundance of bacterial phylum and genera was assessed across all experimental groups, including Kk, Km, Kkm, and CK under both saline and nonsaline conditions. Comparative analyses between Kkm‐SA group and the individual inoculation groups (Kk‐SA and Km‐SA) revealed distinct shift in microbial community composition Supporting Information S1: Figure S5A,B). Specifically, Kkm‐SA exhibited a prominent enrichment of both *Bacillus* and *Kushneria*, a pattern less evident in the Kk‐SA and Km‐SA treatment. These findings suggest a potential synergistic interaction between the two *Kushneria* isolates when co‐inoculated under salt stress conditions. Although Kk‐SA and Km‐SA also displayed compositional differences compared to the uninoculated salt‐stressed control (CK‐SA), these shifts were less substantial than those observed in Kkm‐SA.

Linear discriminant analysis (LDA) confirmed *Bacillus* and *Kushneria* as key discriminatory taxa in the Kkm‐SA group (Figure 7D), further supporting their dominant role in shaping a beneficial rhizosphere under saline conditions. Supporting Information S1: Figure S5C also confirmed that both taxa were significantly more abundant in Kkm‐SA than in Kk‐SA or Km‐SA, implying that the consortium creates a unique microbiome environment favourable for plant growth and stress resilience.

Beyond *Bacillus* and *Kushneria*, other notable taxa were observed in different treatment groups, as indicated by LDA in Supporting Information S1: Figure S5C. Specifically, Kk‐SA was associated with an increase in *Cytophagales*, *Sphingobium*, and *Caulobacteraceae*, reflecting a distinct microbial composition. Conversely, Km‐SA was enriched *Epibacterium*, *Weeksellaceae*, and *Taibaiella*. These results indicate that while individual strains influenced microbial profiles in distinct ways, the Kkm consortium exerted a more unified and impactful restructuring effect, particularly by promoting the co‐dominance of *Bacillus* and *Kushneria* under salinity.

The redundancy analysis (RDA) using the top 20 genera explained 36.5% and 24.39% of the total variance along the first and second axes, respectively. Among the most abundant genera, *Bacillus* was closely aligned with the vectors representing SOD, POD, and chlorophyll (CHL), indicating a strong positive association with antioxidant enzyme activity and photosynthetic capacity. These results suggest that the abundance of *Bacillus* is positively correlated with enhanced antioxidant defence mechanisms and maintained photosynthetic performance under salt stress conditions, indicating its potential role in plant salt stress tolerance (Figure 7E). This correlation underscores the potential of *Bacillus* to improve plant health and productivity under Kkm‐SA treatment. The synergistic relationship between *Kushneria* and *Bacillus* likely enhances rhizosphere colonisation, biofilm formation, and nutrient uptake, thus facilitating better plant growth under salt‐stress conditions. These results highlight how combining salt‐tolerant *Kushneria* isolates can reshape the rhizosphere microbiome, creating a more favourable environment that significantly enhances plant resilience and growth.

Collectively, these findings show that the Kkm consortium, particularly under salt stress (Kkm‐SA), reshapes the rhizosphere microbiome more effectively than individual strains or controls. This restructuring is characterised by the selective enrichment of *Bacillus* and *Kushneria*, which are closely associated with key plant‐beneficial traits such as stress resilience, antioxidant activity, and photosynthetic performance. The results highlight the value of microbial consortia derived from halophytic environments as promising bioinoculants to enhance crop productivity in saline soils.

### 2.9 Synergistic Interactions Between *Kushneria* and *Bacillus* Species Under Saline Conditions

**Figure 8:**
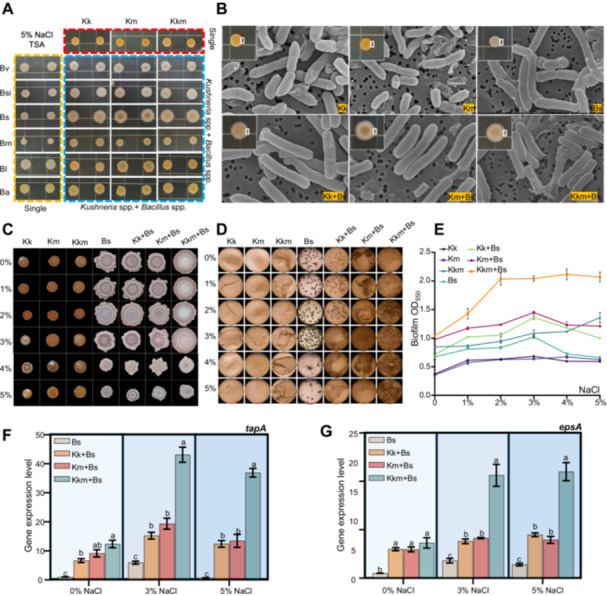
Enhanced biofilm formation through cocultivation of *Kushneria* isolates and *Bacillus* spp. under saline conditions. (A) Altered colony morphology of various *Bacillus* strains cocultured with *Kushneria* isolates (Kk, Km, Kkm) on TSA containing 5% NaCl. Strains include Bv (*B. velezensis*), Bsi (*B. siamensis*), Bs (*B. subtilis*), Bm (*B. megateium*), Bl (*B. licheniformis*), and Ba (*B. altitudinis*). (B) Scanning electron microscopy (SEM) image showing the outer edge of mixed colonies. (C) Biofilm formation on TSA supplemented with glycerol and MnSO_4_. (D–E) Quantitative assays of biofilm production in TSB medium. (F–G) Gene expression analysis of biofilm‐associated *Bacillus* genes, *tapA* and *epsA*, under Cocultivation conditions. [Color figure can be viewed at wileyonlinelibrary.com]

To explore whether *Kushneria* isolates can promote the abundance or activity of *Bacillus* species under saline conditions, we performed cocultivation experiments with various *Bacillus* strains alongside *Kushneria* isolates (Kk, Km or Kkm). This approach led to diverse changes in the size and colour of *Bacillus* colonies, with *Bacillus subtilis* exhibiting the most pronounced response (Figure 8A). We selected *B. subtilis* (Bs) for further study to determine whether the external regions of the mixed colonies were composed of *Kushneria* isolates or *Bacillus*. Utilising scanning electron microscopy (SEM) and 16S rRNA sequencing, we confirmed that these external regions were predominantly *Bacillus* (Figure 8B). Notably, colonies treated with Kkm (Kkm + Bs) displayed a substantial amount of a viscous extracellular matrix, suggesting a strong biofilm‐forming capacity. In contrast, samples treated with Km (Km + Bs) exhibited a moderate amount of extracellular matrix, indicating a less pronounced biofilm formation, and Kk‐treated (Kk + Bs) colonies showed no significant extracellular matrix accumulation. These findings suggest a synergistic interaction between Kkm and *Bacillus* that may promote biofilm formation, prompting us to conduct further biofilm assays.

To further investigate this synergy, biofilm assays were performed on tryptic soy agar (TSA) supplemented with 0.1% glycerol and 10 µM MnSO_4_, which are known to stimulate biofilm formation in *Bacillus*. The results showed that Kkm+Bs co‐cultures significantly enhanced biofilm development, with maximal formation observed at 3% NaCl (Figure 8C). Quantitative biofilm analysis using tryptic soy broth (TSB) revealed similar trends. The Kkm+Bs treatment achieved peak biofilm biomass at 2% NaCl and maintained high levels even up to 5% NaCl (Figure 8D,E). Although coculture with Kk or Km also enhanced biofilm production compared to Bs alone, their effects were notably weaker than the Kkm+Bs combination. To support these phenotypic observations, we analyzed the expression of two *Bacillus* biofilm‐related genes, *tapA* and *epsA*. qRT‐PCR results showed that all co‐cultures (Kk+Bs, Km+Bs, and Kkm+Bs) significantly upregulated these genes relative to control, with the highest expression levels observed in the Kkm+Bs treatment (Figure 8F,G). This suggests that the Kkm consortium strongly induces biofilm regulatory pathways in *Bacillus*.

To determine whether direct microbial contact is essential for the observed synergy, we performed additional coculture assays using cell‐free supernatants from *Kushneria* cultures grown in 5% NaCl. When *Bacillus* was incubated with these supernatants under 1% NaCl, a modest increase in biofilm formation was observed. However, at higher salinity (5% NaCl), this effect diminished and became statistically insignificant (Supporting Information S1: Figure S6A,B). These results indicate that while soluble metabolites may contribute slightly under low‐salt conditions, direct physical interactions between *Kushneria* and *Bacillus* are critical for maintaining robust synergistic effects under salt stress.

## 3 Discussion

As global warming exacerbates soil salinization, exploring salt‐tolerant plant growth‐promoting bacteria (St‐PGPB) is becoming increasingly important for sustainable agricultural practices (Ansari et al. 2019; Meinzer et al. 2023; Singh et al. 2015). This study highlights the significant potential of halophyte‐derived bacteria, *K. konosiri* and *K. marisflavi*, in promoting plant growth and enhancing salinity tolerance. To evaluate the effectiveness of halophyte‐derived bacteria across different plant systems, we employed *Arabidopsis thaliana* as a model plant for mechanistic insights and *Brassica rapa* (cabbage) as a representative crop to assess translational potential under agriculturally relevant conditions. This dual‐system approach allowed us to examine both fundamental responses and practical applications of microbial inoculation under salinity stress. Our findings show that both Kk and Km, individually or co‐inoculated as a consortium (Kkm), effectively enhance the salinity tolerance of *Arabidopsis* seedlings and cabbage. The mechanisms behind this enhancement appear to include increased phytohormone production, heightened antioxidant activity, and modulation of the rhizosphere microbial community, all of which contribute to plant resilience under saline conditions. Notably, the synergistic effects were more pronounced in soil‐based system than in agar plate assay, indicating that complex microbial interactions in the rhizosphere amplify the plant‐beneficial outcome beyond direct microbe‐plant contact. To our knowledge, this is the first to elucidate a synergistic interaction between *Kushneria* and *Bacillus* species that enhance biofilm formation and promote rhizosphere colonisation under salt stress. This novel finding offers mechanistic insight into how co‐inoculated halophilic strains cooperatively restructure the rhizosphere microbial community and boost the expression of plant‐beneficial traits, laying a foundation for consortium‐based bioinoculant strategies in saline agriculture.

From the halophyte *Suaeda maritima*, we isolated 96 bacterial strains, with the *Kushneria* genus representing 40.6%, primarily Kk (25.0%) and Km (15.6%). These strains exhibited extreme halotolerance, surviving NaCl concentrations up to 25% (Figure 1C), consistent with their natural halophytic origin (Duan et al. 2022). While previous studies have reported PGP traits of *Kushneria*, they have largely focused on individual strains, leaving the potential of strain combinations underexplored. Cocultivation of Kk and Km significantly enhanced key PGP traits, including increased production of IAA, proline, and exopolysaccharide (EPS), and improved phosphate solubilisation (Figure 1D–H). These compounds play vital roles in mitigating salt stress, as IAA modifies root architecture, proline acts as an osmoprotectant and antioxidant, and EPS protects root surfaces (Fatima and Arora 2021; Kadmiri et al. 2018; Wang et al. 2015). In *Arabidopsis* plate assays, all inoculated treatments improved growth under saline conditions. Although Kkm did not outperform single strains in biomass accumulation (Figure 2), IAA accumulation was highest in Kkm‐treated roots, indicating early synergistic effects that may be more pronounced in complex soil environments. The absence of synergy in agar assays likely reflects their simplicity, which cannot replicate the dynamic interactions of the rhizosphere.

A key mechanism contributing to salt tolerance in *Kushneria*‐inoculated plants is the regulation of sodium (Na⁺) uptake. Treatments with *Kk*, *Km* and the *Kkm* consortium significantly reduced Na⁺ accumulation in plant tissues and enhanced the K⁺/Na⁺ ratio (Figure 3A–E), a critical factor in maintaining ionic homoeostasis under salt stress (Metoui Ben Mahmoud et al. 2020; Panwar et al. 2016). Sodium (Na⁺), chloride (Cl⁻), and other major cations play essential roles in plant responses to salinity; thus, accurate quantification of these ions is vital for a comprehensive understanding of ionic balance and salt‐induced physiological stress. While this study focused primarily on physiological traits and the microbiome‐mediated effects of *Kushneria* inoculation, the absence of detailed mineral nutrient profiling, particularly measurements of chloride (Cl⁻), represents a limitation. Future research should address this aspect to provide a more complete picture of ion dynamics under salinity stress.

Another significant finding is the enhanced antioxidant activity in *Arabidopsis* treated with *Kushneria* isolates. Under salt stress, reactive oxygen species (ROS) accumulation can cause severe oxidative damage to plant cells (Rahman et al. 2021; Talaat and Shawky 2014). Remarkably, our results showed that Kk, Km and Kkm treatments reduced ROS levels and increased the activities of antioxidant enzymes such as POD and SOD (Figure 4D,E). POD activity increased by 1.4‐fold and SOD activity by 1.6‐fold in Kkm‐treated plants compared to salt‐stressed controls, indicating a robust activation of the antioxidant defence system. This antioxidant response is essential for mitigating oxidative stress, a common mechanism by which PGPB enhance plant resilience to abiotic stress (Hafez et al. 2021). The enhanced antioxidant capacity may also be linked to the increased proline content observed in inoculated plants, as proline serves dual roles as an osmoprotectant and ROS scavenger (Figure 4F).

Under varying salt concentrations (0%, 3%, 6% and 9%), Kkm significantly enhanced biofilm production, with the strongest effect observed at 3% NaCl. At this concentration, Kkm increased biofilm formation by 1.5‐fold compared to Kk and 1.9‐fold compared to Km (Figure 1F). Since biofilm formation is essential for microbial colonisation (Ansari and Ahmad 2018; Primo et al. 2015), we hypothesised that Kkm may perform better in soil‐based experiments. Biofilms provide a protective microenvironment that enhances bacterial survival under stress conditions and facilitates the establishment of beneficial plant‐microbe interactions through concentrated delivery of metabolites and signalling molecules (Kumar et al. 2017; Parrilli et al. 2022).

In soil with cabbage under salt stress (Figure 5), these strains dramatically promoted growth, increasing biomass, shoot and root fresh weights, and chlorophyll content (Figure 5C,D,F,G,I). Compared to salt‐stressed controls, Kkm‐treated plants showed marked improvements in biomass and chlorophyll accumulation, with increases of 1.84‐fold in shoot fresh weight, 1.50‐fold in shoot dry weight, 1.34‐fold in root fresh weight, 1.35‐fold in root dry weight, and 1.52‐fold in chlorophyll content. Notably, these values were also significantly higher than those observed in plants treated with KK or KM alone, highlighting the synergistic effect of the combined inoculation. These results suggest that Kkm treatment effectively alleviated salt‐induced growth inhibition and protected the photosynthetic apparatus. PCA analysis further showed that the Kkm group formed a distinct cluster along the PC1 axis under salt stress, supporting our hypothesis that co‐inoculation creates unique physiological states in plants that differ qualitatively, not just quantitatively, from single‐strain inoculation.

Additionally, cabbage plants inoculated with *Kushneria* strains showed reduced Na⁺ accumulation and increased K⁺ content in leaf tissues, resulting in a significantly improved K⁺/Na⁺ ratio under salt stress. The K⁺/Na⁺ ratio in Kkm‐treated plants was significantly higher than in plants treated with individual strains, approaching that of non‐stressed controls. This ion balance was particularly notable in the Kkm treatment, which performed better than individual strains. At the biochemical level, antioxidant enzyme activities including POD and SOD were also substantially enhanced in inoculated cabbage plants, along with increased proline content and reduced MDA levels, further confirming the role of *Kushneria* strains in mitigating salt‐induced oxidative damage (Figure 5K, Supporting Information S1: Table S3).

Rhizosphere sequencing revealed that Kkm dramatically altered microbial diversity and structure under salt stress. Alpha and beta diversity analyses showed that Kkm‐SA treatments clustered distinctly from other groups, indicating a unique microbial composition (Supporting Information S1: Figures S4). RDA showed a strong correlation between *Bacillus* abundance and antioxidant activity and chlorophyll levels, suggesting its functional role in stress mitigation (Figure 7E). LDA analysis confirmed a higher relative abundance of *Kushneria* and *Bacillus* in Kkm‐SA‐treated rhizospheres (Supporting information S1: Figure S5C), with *Bacillus* species well known for their role in salinity tolerance (Han et al. 2014; Kumar et al. 2021; Yoo et al. 2019).

To understand the interaction between Kkm and *Bacillus*, coculture assays were conducted. Among several strains, *B. subtilis* showed the strongest interaction, forming larger, matrix‐rich colonies in the presence of Kkm (Figure 8A,B). Biofilm assays confirmed that Kkm + Bs co‐cultures produced significantly more biofilm than Bs alone, particularly under 2%–5% NaCl (Figure 8C–E). Expression of *tapA* and *epsA*, key genes in *Bacillus* biofilm formation, was significantly upregulated in Kkm+Bs cultures under high‐salt stress (Figure 8F,G). These effects were only partially reproduced using *Kushneria* supernatants, suggesting that direct contact is necessary to sustain strong synergistic interactions under saline conditions (Supporting Information S1: Figure S6A,B). These findings highlight the role of physical interactions in facilitating beneficial microbe‐microbe and microbe‐plant relationships in the rhizosphere.

This study provides robust evidence that co‐inoculation with *K. konosiri* and *K. marisflavi* (Kkm) offers multiple synergistic benefits for plant growth and salt stress tolerance. These include enhanced phytohormone production, reduced oxidative damage, improved ion regulation, and significant modulation of the rhizosphere microbiome. The demonstrated synergy between *Kushneria* and *Bacillus*, particularly in promoting biofilm formation and root colonisation under salt stress, underscores the potential of halophyte‐derived microbial consortia as effective bioinoculants. These findings lay a scientific foundation for the development of next‐generation microbial solutions for sustainable agriculture in salt‐affected soils, a timely approach in the face of increasing soil salinity and global food security challenges.

## 4 Materials and Methods

### 4.1 Sample Collection and Bacterial Isolation

Plant specimens, including leaves, stems, and roots, were collected from the halophyte *Suaeda maritima* growing on a beach in Saemangeum, Gimje‐si, Jeollabuk‐do, Republic of Korea (35°50'21.012″ N, 126°35'12.588″ E). The plant tissues were surface sterilised by immersion in 1% (v/v) sodium hypochlorite solution for 10 min, followed by five rinses with sterile water. The sterilised tissues were then homogenised using a blender, and the homogenate was serially diluted. The dilutions were plated on tryptic soy agar (TSA) medium and incubated at 25°C for 7 days. For the salt‐tolerance assay, bacterial isolates were incubated on TSA medium supplemented with NaCl concentrations ranging from 0% to 30% (w/v) for 3 days at 25°C.

### 4.2 Identification of Bacterial Isolates

The bacterial isolates were identified through 16S rRNA gene analysis. Universal primers 27F (5′‐AGAGTTTGATCCTGGCTCAG‐3′) and 1492R (5′‐GGTTACCTTGTTACGACTT‐3′) were used for PCR amplification, along with primers 518F (5′‐CCAGCAGCCGCGGTAATAC‐3′) and 805R (5′‐GACTACCAGGGTATCTAATC‐3′) (Hiergeist et al. 2016). The sequences obtained were analyzed using the BLAST tool available at the National Center for Biotechnology Information (NCBI; https://blast.ncbi.nlm.nih.gov/Blast.cgi). Phylogenetic relationships were determined using the neighbour‐joining (NJ) method (Saitou and Nei 1987).

### 4.3 Biochemical Characterisation of *Kushneria* Isolates

Detailed experimental procedures for assessing the biochemical properties of *Kushneria* isolates, including IAA production, proline synthesis, biofilm formation, phosphate solubilisation, siderophore production, EPS production, catalase activity, proteolytic activity, and amylase activity, are provided in the Supporting Materials. Each isolate (Kk and Km) was adjusted to an optical density at 600 nm (OD₆₀₀) of 0.1 before use. For a bacterial consortium composed of two *Kushneria* species (Kkm), was prepared by mixing equal volumes of each isolate, following OD₆₀₀ adjustment, to achieve a final OD₆₀₀ of 0.1.

### 4.4 Plant Material Preparation and Growth Assays

Seeds of *Arabidopsis thaliana* DR5::GUS transgenic plants (Ulmasov et al. 1997) with a Col‐0 background were surface‐sterilised in a 1.3% hypochlorite solution for 10 min, followed by rinsing with sterile double‐distilled water (ddH₂O). The sterilised seeds were stratified at 4°C for 48 h and then grown on Murashige and Skoog (MS) medium under a 16‐h light/8‐h dark cycle for 7 days. For salinity stress treatment, 7‐day‐old *Arabidopsis* seedlings were transferred to 1/2 MS medium supplemented with varying concentrations of NaCl. The plates were positioned vertically and incubated under a 16 h light/8 h dark cycle for 14 days. For the bacterial treatment group, fresh bacterial cultures grown for 3 days were adjusted to an OD_600_ of 0.1, and 10 μL of the bacterial suspension was applied to the TSA medium overlaid on the MS medium. The control group received only water treatment. Each experimental set comprised seven seedlings per plate, with three replicated plates per group. The entire experiment was conducted five times to ensure reliability. Consistent plant growth conditions were maintained throughout the experiments. For the bacterial consortium (Kkm), equal volumes of Kk and Km cultures were combined at a 1:1 ratio to maintain the same final OD₆₀₀ of 0.1.

Cabbage seeds (*Brassica rapa* subsp. *pekinensis* (Lour.) Hanelt) were sown in 70 g of commercial horticultural substrate soil (Hanpanseung, Shinhwa GreenTech Co., Korea) and grown under controlled environment conditions (22°C, 16‐h light/8‐h dark photoperiod). After 7 days, seedlings were thinned to retain one plant per pot. On Days 8 or 9, 10 mL of bacterial solution (OD_600_ = 0.5) was applied to designated treatment group. For the consortium treatment (Kkm), strains Kk and Km were mixed at a 1:1 ratio to achieve a final OD_600_ of 0.5. Control plant received in 10 mL of tryptic soy broth (TSB) instead. After two rounds of bacterial inoculation, salt stress was initiated on day 10. Seedlings were irrigated with 10 mL of 200 mM NaCl solution every 3 days, while the control group received an equivalent volume of water. After seven rounds of salt treatment across 22 days, plants were harvested on Day 32. Measurement taken included fresh weight, root weight, and chlorophyll content. Each treatment was tested with at least 12 seedlings per group, and the experiment was independently repeated (Figure 5B).

### 4.5 Chlorophyll Content Measurement

Leaf chlorophyll concentration in *Arabidopsis* and cabbage was measured using the Lorenzen Cj (1967). Absorbance of the supernatant obtained from ethanol‐infiltrated leaf tissues was recorded at 645 nm and 663 nm. The total chlorophyll content was calculated using the following formula:

$$\text{Total C}h\text{lorop}h\text{yll}\;(\mu g/\text{mg})=\frac{\left(20.2\;x\;D_{645}+8.02\;{x\;D}_{663}\right)}{\mathrm{Fresh weight}\;(\mathrm{mg})}$$

### 4.6 β‐Glucuronidase (GUS) Histochemical Staining

To detect auxin levels in plant tissues, the method described by Gallagher (2012) was used. Seven‐day‐old *Arabidopsis* seedlings grown on 1/2 MS medium supplemented with 0, 100 or 150 mM NaCl and cocultured with bacteria for 14 days were stained with a GUS solution containing 1 mM X‐Gluc (GOLDBIO, CAT: G1281C1) and incubated at 37°C for 24 h. The stained roots were observed under a Nikon SMZ1270 microscope (0.63x).

### 4.7 Fluorescence Microscopy

Ten‐day‐old *Arabidopsis* seedlings were treated with 200 mM NaCl for 24 h, both in the presence and absence of bacterial Cocultivation. Intracellular Na⁺ accumulation in roots was assessed following the method of Choi et al. (2011), using 10 mM CoroNa‐Green (Invitrogen, Thermo Fisher Scientific) for staining. Intracellular ROS levels were measured using 5 μM DCFH‐DA (Cayman Chemical Company) as per the protocol provided by Katiyar‐Agarwal et al. (2006). Fluorescence intensities were analyzed using Image J software (Bankhead 2014).

### 4.8 Assessment of Antioxidant Ability in Plants

Fresh shoots of *Arabidopsis* and cabbage were rapidly frozen in liquid nitrogen, ground, and homogenised in 100 mM phosphate‐buffered saline (PBS) buffer (pH 7.8). The homogenate was centrifuged at 4°C to obtain a crude enzyme extract, and protein concentration was quantified using a Multiskan SkyHigh microplate reader.

POD activity was determined as described in reference (Chen and Zhang 2016). The assay mixture included 100 μL of crude enzyme extract, 50 μL of 20 mM guaiacol, 30 μL of 30% H₂O₂, and 3.0 mL of 100 mM phosphate buffer (pH 7.0). Absorbance at 470 nm was measured immediately and monitored every 15 s for 3 min. POD activity was calculated using following the formula:

$$\text{POD activity }(U/\text{mg protein})=△A_{470\;}x\;({V/V}_{t})/(0.01\;x\;t)/\mathrm{Cp}$$

SOD activity was assessed using the method of Chen and Zhang (2016). The reaction mixture consisted of 1.3 mL of 50 mM sodium carbonate buffer (pH 10.2), 500 μL of 96 μM nitro blue tetrazolium chloride (NBT), and 100 μL of 0.6% Triton X‐100. The reaction was initiated by adding 20 mM hydroxylamine hydrochloride (pH 6.0), followed by 70 μL of crude enzyme extract. Absorbance at 560 nm was measured every 15 s for 3 min. SOD activity activity was assessed using the method of Chen anda:

$$\text{SOD activity}\;(U/\text{mg protein})=[(A_{\mathrm{ck}}-A_{s})xV]/(0.5x\;A_{\mathrm{ck}}x\;V_{t})/\mathrm{Cp}$$

Proline concentration in seedlings was determined using the acid‐ninhydrin method by Bates et al. (1973) with absorbance measured at 520 nm after extraction with toluene. Malondialdehyde (MDA) content in seedlings was measured using the Abcam lipid peroxidation assay kit, following the manufacturer's instructions.⁺ content were measured according to Iseki et al.

### 4.9 Determination of Na^+^ and K^+^ Content in Plant Tissues

Na⁺ and K⁺ content were measured according to Iseki et al. (2017). Seven‐day‐old *Arabidopsis* seedlings were treated under saline (150 mM NaCl) and nonsaline conditions with bacterial strains Cocultivation for 7 days. Cabbage seedlings were treated as described above. After treatment, plants were dried at 60°C until a constant weight was achieved. The dried material was dissolved in deionized water, and the ion content of Na⁺ and K⁺ in the supernatant was measured using a LAQUA twin ion metre (Horiba, Japan).

### 4.10 RNA Extraction and qRT‐PCR Gene Expression Analysis

Ten‐day‐old *Arabidopsis* seedlings were co‐cultivated with *Kushneria* strains (OD₆₀₀ = 0.1). For the bacterial consortium (Kkm), strains Kk and Km were combined at a 1:1 ratio to achieve a final OD₆₀₀ of 0.1. The seedlings were then transferred to half‐strength Murashige and Skoog (1/2 MS) liquid medium supplemented with NaCl at concentrations of 0, 100, or 150 mM. Co‐incubation was carried out for 3, 6 or 12 h to assess the effects of salinity and bacterial interaction over time.

Total RNA was extracted from the shoots using the RNAqueous Total RNA Isolation Kit (Thermo Fisher Scientific, Waltham, MA, USA), and any residual genomic DNA was removed using the TURBO DNA‐free Kit (Thermo Fisher Scientific). The purified RNA was then reverse‐transcribed into first‐strand cDNA using the High‐Capacity cDNA Reverse Transcription Kit (Thermo Fisher Scientific). Quantitative real‐time PCR (qRT‐PCR) was performed using the 2× Real‐Time PCR Master Mix Kit (BioFACT Co., Daejeon, Korea), with TUBULIN2 serving as the internal reference gene. Based on previous studies (Baek et al. 2019), the salt‐responsive gene *RD29A*, RD20, and *KIN1* were selected for expression analysis. PCR amplification was carried out on a CFX Connect Real‐Time PCR Detection System (BIO‐RAD, Berkeley, CA, USA) under the following thermal cycling conditions: an initial denaturation at 95°C for 15 min; followed by 40 cycles of denaturation at 95°C for 20 s, annealing at 58°C for 30 s, and extension at 72°C for 15 s; with final extension at 72°C for 7 min. Primer sequences are listed in supporting information Table S1. Each reaction was performed, with three biological replicates and three technical replicates per sample.

### 4.11 Metagenomic DNA Extraction and Sequencing

Rhizosphere soil samples were collected from the roots of plants used in the pot assay, retaining approximately 1 mm of soil on the root surfaces. These samples were immersed in phosphate‐buffered saline (PBS) and subjected to ultrasonication for 3 min to detach the rhizosphere soil. Metagenomic DNA was extracted using the DNeasy PowerSoil Kit (Qiagen, Hilden, Germany) following the manufacturer's protocol. The extracted DNA was quantified using the Quant‐IT PicoGreen assay (Invitrogen).

For sequencing, the purified DNA was further quantified using quantitative PCR (qPCR) as per the qPCR Quantification Protocol Guide (KAPA Library Quantification Kits for Illumina Sequencing platforms) and assessed for quality using the TapeStation D1000 ScreenTape system (Agilent Technologies, Waldbronn, Germany). Paired‐end sequencing (2 × 300 bp) was conducted by Macrogen (Seoul, South Korea) using the Illumina MiSeq platform (Illumina, San Diego, USA). The V3–V4 region of the bacterial 16S rRNA gene was amplified using barcoded primers 341F (CCTACGGGNGGCWGCAG) and 805R (GACTACHVGGGTATCTAATCC).

### 4.12 Metabarcoding Sequencing Processing and Analysis of Data

Following sequencing, raw data were processed to generate paired‐end FASTQ files, classified using index sequences. Sequencing adaptors and primers were removed using Cutadapt (v3.2) (Martin 2011). Sequence processing and error correction were performed using the DADA2 pipeline (v1.18.0) in R (v4.0.3, https://www.R-project.org/) (Callahan et al. 2016). Sequences were trimmed to 250 bp (forward) and 200 bp (reverse), excluding those with more than two expected errors. Batch‐specific error model filtered noise, and chimeric, sequences were removed to generate Amplicon Sequence Variants (ASVs).

Taxonomic classification of ASVs was carried out using BLAST+ (v2.9.0) against the NCBI 16S Microbial Database, with a threshold of 85% query coverage and identity (Camacho et al. 2009). Multiple sequence alignments were performed using Mafft (v7.475) (Katoh and Standley 2013), and a phylogenetic tree was constructed using FastTreeMP (v2.1.10) (Price et al. 2010).

Comparative analysis of microbial communities was conducted using QIIME (v1.9) (Caporaso et al. 2010). Alpha diversity metrics, including the Shannon index and Inverse Simpson index, were calculated to assess species diversity and evenness within the samples. Rarefaction curves and Chao1 values were generated to evaluate the adequacy of sequencing depth. Beta diversity was assessed based on Weighted and Unweighted UniFrac distances, and the relationships among samples were visualised using PCA in STAMP (v2.1.3) (Parks et al. 2014).

### 4.13 Investigation of Synergistic Interaction Between *Kushneria* Isolates and *Bacillus* spp

#### 4.13.1 Cocultivation and Colony Morphology Observation

To investigate potential interactions among *Kushneria* isolates (Kk, Km, Kkm), and genus *Bacillus* members, cocultivation experiments were conducted on TSA supplemented with 5% NaCl. Individual cultures of each bacterium were initially grown separately. Each bacterial strain, including various *Bacillus* species and *Kushneria* isolates, (Kk, Km, Kkm) was adjusted to an OD_600_ = 0.1 in liquid media. Standardised suspensions were then mixed and 10 μL aliquots of each mixture were spotted onto TSA plates with 5% NaCl for cocultivation. Morphological changes in colony size and colour were observed, with *B. subtilis* showing the most significant response. Scanning electron microscopy (SEM) and 16S rRNA sequencing analyzed the composition surrounding the colonies, confirming that *Bacillus* species primarily populated these regions in the Kkm+Bs co‐cultures, indicating enhanced biofilm formation potential.

#### 4.13.2 Plate Visualisation of Biofilm Formation

Biofilm formation was visualised on TSA‐GM plates (Tryptic Soy Agar supplemented with 0.1% glycerol, 10 μM MnSO₄, 40 mg/L Congo red, and 20 mg/L Coomassie brilliant blue), with modifications to the protocol described by (Chen et al. 2024; Yang et al. 2022). Overnight‐grown bacteria cultures were adjusted to an OD₆₀₀ of 0.1, and 10 μL of the bacterial suspension was spotted onto the TSA‐GM plates, followed by incubation at 30°C for 3 days. Biofilm formation was documented via photography.

#### 4.13.3 Liquid Biofilm Formation Experiment and Quantitative Testing

For liquid biofilm assays, 48‐well plates were prepared with 500 μL of TSB (Tryptic Soy Broth) containing varying salt concentrations per well. Plates were incubated statically at 30°C for 7 days. Biofilms were stained with 0.1% crystal violet (Kamimura et al. 2022). Excess dye was removed, and biofilm biomass was quantified by solubilizing the bound dye in ethanol and measuring the OD₅₅₀ with a microplate reader.

#### 4.13.4 qRT‐PCR Analysis of Biofilm‐Related Genes

Total bacterial RNA was extracted RNAqueous total RNA isolation kit (Thermo Fisher Scientific, Waltham, MA, USA), and genomic DNA contamination was removed using the TURBO DNA‐free kit (Thermo Fisher Scientific, Waltham, MA, USA). Complementary DNA (cDNA) synthesis and subsequent quantitative real‐time PCR (qRT‐PCR) analysis were performed using high‐capacity cDNA reverse transcription Kit (Thermo Fisher Scientific, Waltham, MA, USA). Two *Bacillus* genes pivotal to biofilm formation, *tapA* and *epsA*, were selected for investigation based on prior research.

The qRT‐PCR reactions were conducted with the 2 × Real‐Time PCR Master Mix Kit (BioFACT Co., Daejeon, Korea), employing BS‐16S expression as an endogenous control. PCR amplifications were performed under the following conditions using a CFX Connect Real‐Time PCR Detection System (BIO‐RAD, California, Berkeley, USA): initial denaturation at 95°C for 15 min, followed by 40 cycles of denaturation at 95°C for 20 s, annealing at 55°C for 20 s, and extension at 72°C for 20 s.

Primer information for qRT‐PCR experiment is provided in Supporting Information S1: Table S1. Each condition was replicated biologically three times, with technical triplicates for each sample.

### 4.14 Statistical Analysis

Statistical analyses were performed using GraphPad Prism 9. Group differences were assessed using *t*‐tests and one‐way ANOVA, with statistical significance set at *p* ≤ 0.05. Quantitative real‐time PCR (qRT‐PCR) analyses were performed in triplicate, and the average values of 2^‐ΔCT^ were used to evaluate gene expression differences.

## Conflicts of Interest

The authors declare no conflicts of interest.

## Supporting information


**Figure S1:** Growth curves of *Kk*, *Km*, and *Kkm* strains under different NaCl concentrations. Bacterial cultures were incubated in media containing 0%, 3%, 6%, or 9% NaCl, and growth was monitored by measuring optical density at 600 nm (OD_600_) over a 72‐hour period. Each point represents the mean ± standard deviation of three biological replicates. **Figure S2:** Expression levels of salt stress‐responsive genes in *Arabidopsis* seedlings following *Kushneria* inoculation under different salt concentrations. Relative expressions of *RD29A*, *RD20*, and *KIN1* in *Arabidopsis* shoots treated with Kk, Km, and Kkm under 0, 100, and 150 mM NaCl conditions at 3 h, 6 h, and 12 h post‐treatment. Expression levels were normalized to the endogenous control gene *TUBULIN2* (*TUB2*), and values are presented relative to the untreated control (CK). Bars represent mean ± SD of three independent biological replicates. A broken Y‐axis was applied to allow comparison across genes with different expression magnitudes. **Figure S3:** Biofilm formation and growth dynamics of *Kushneria* spp. (A) Biofilm formation by *Kushneria* spp. in solid and liquid media. The top row displays biofilms formed on plates, the middle row shows biofilms in liquid culture, and the bottom row presents disturbed liquid cultures. (B) Growth curve of *Kushneria* spp. under different treatments, measured by OD_600_ over a 48‐hour period. **Figure S4:** Microbial community diversity and composition across treatments under non‐saline (DW) and saline (SA) conditions. (A) Shannon diversity index showing significant differences in microbial diversity among treatments (CK, Kk, Km, and Kkm) under DW and SA conditions.(B) Richness analysis comparing microbial diversity across treatments. (C) Pielou's evenness index (Robbins) assessing community evenness. (D) Principal Component Analysis (PCA) of beta‐diversity, illustrating variations in microbial composition. (E) Phylogenetic tree depicting microbial community clustering across samples, with a branch scale of 0.02. Significance levels: *p < 0.05, *p < 0.01, *ns = not significant*. **Figure S5:** Comparative analysis of microbial community composition and differentially enriched taxa across treatments. (A) Relative abundance of microbial communities at the phylum level. (B) Relative abundance of microbial communities at the genus level. (C) LEfSe analysis highlighting significantly enriched genera across different treatments (CK, Kk, Km, and Kkm) under both DW and SA conditions. **Figure S6:** Effect of *Kushneria* supernatant on *Bacillus subtilis* biofilm formation under different NaCl concentrations. (A) Representative images depicting biofilm formation by *B. subtilis* alone (Bs) and in co‐culture with *K. konosiri* supernatant (KkS + Bs), *K. marisflavi* supernatant (KmS + Bs), and the combined supernatant (Kkms + Bs) under 1%, 2%, 3%, 4%, and 5% NaCl conditions. (B) Quantitative analysis of biofilm biomass (OD_550_) across different treatments, demonstrating the influence of *Kushneria* supernatants on *B. subtilis* biofilm formation at varying salt concentrations. **Table S1:** Primer list for qRT‐PCR in this study. **Table S2:** Summary of two‐way ANOVA showing the effects of salt stress, bacterial treatment, and their interaction on physiological traits in *Arabidopsis* and cabbage. Significance levels: ns, not significant (P ≥ 0.05); * P < 0.05; ** P < 0.01; *** P < 0.001; **** P < 0.0001. **Table S3:** Effects of *Kushneria* treatment on ion content, antioxidant enzyme activities, proline, and MDA in plants under salt‐stressed and non‐stressed conditions. Different letters indicate statistically significant differences between bacteria‐treated and untreated groups, based on two‐way ANOVA (p < 0.05).

## Data Availability

Raw amplicon sequencing data have been deposited in the Sequence Read Archive (SRA). The accession numbers are as follows: CK‐DW (SRR29841587, SRR29841586, SRR29841575), CK‐SA (SRR29841570, SRR29841569, SRR29841568), Kk‐DW (SRR29841567, SRR29841566, SRR29841565), Kk‐SA (SRR29841564, SRR29841585, SRR29841584), Km‐DW (SRR29841583, SRR29841582, SRR29841581), Km‐SA (SRR29841580, SRR29841579, SRR29841578), Kkm‐DW (SRR29841577, SRR29841576, SRR29841574), Kkm‐SA (SRR29841573, SRR29841572, SRR29841571).
