# Supplementary material for: Halophyte‐Derived Kushneria Strains Enhance Salt Tolerance and Rhizosphere Dynamics in Cabbage
Source: Plant Cell Environ. 2025 Oct 12;49(1):531–49. doi: 10.1111/pce.70234 (PMC12675972; doi:10.1111/pce.70234)
Supplement: Supplementary file 1 — Figure S1: Growth curves of Kk, Km, and Kkm strains under different NaCl concentrations. Bacterial cultures were incubated in media containing 0%, 3%, 6%, or 9% NaCl, and growth was monitored by measuring optical density at 600 nm (OD600) over a 72‐hour period. Each point represents the mean ± standard deviation of three biological replicates. Figure S2: Expression levels of salt stress‐responsive genes in Arabidopsis seedlings following Kushneria inoculation under different salt concentrations. Relative expressions of RD29A, RD20, and KIN1 in Arabidopsis shoots treated with Kk, Km, and Kkm under 0, 100, and 150 mM NaCl conditions at 3 h, 6 h, and 12 h post‐treatment. Expression levels were normalized to the endogenous control gene TUBULIN2 (TUB2), and values are presented relative to the untreated control (CK). Bars represent mean ± SD of three independent biological replicates. A broken Y‐axis was applied to allow comparison across genes with different expression magnitudes. Figure S3: Biofilm formation and growth dynamics of Kushneria spp. (A) Biofilm formation by Kushneria spp. in solid and liquid media. The top row displays biofilms formed on plates, the middle row shows biofilms in liquid culture, and the bottom row presents disturbed liquid cultures. (B) Growth curve of Kushneria spp. under different treatments, measured by OD600 over a 48‐hour period. Figure S4: Microbial community diversity and composition across treatments under non‐saline (DW) and saline (SA) conditions. (A) Shannon diversity index showing significant differences in microbial diversity among treatments (CK, Kk, Km, and Kkm) under DW and SA conditions.(B) Richness analysis comparing microbial diversity across treatments. (C) Pielou's evenness index (Robbins) assessing community evenness. (D) Principal Component Analysis (PCA) of beta‐diversity, illustrating variations in microbial composition. (E) Phylogenetic tree depicting microbial community clustering across samples, with a branch scal [file PCE-49-531-s001.docx]

Supporting information

**Halophyte-Derived *Kushneria* Strains Enhance Salt Tolerance and Rhizosphere Dynamics in Cabbage**

Yuxin Peng ^a^, Ju Huck Lee^a,b^, Cha Young Kim^a^, Jiyoung Lee^a,^ ^b^

^a^Korean Collection for Type Cultures (KCTC), Biological Resource Center, Korea Research Institute of Bioscience and Biotechnology, Jeongeup 56212, Republic of Korea

^b^ KRIBB School of Biotechnology, University of Science and Technology (UST), Yuseong, Daejeon 34113, Republic of Korea

**Correspondences:**

Jiyoung Lee, [jiyoung1@kribb.re.kr](mailto:jiyoung1@kribb.re.kr)

# Supplementary materials

***In-vitro* biochemical characterization of *Kushneria* spp.**

Unless specified otherwise, all bacterial isolates were cultured from fresh 3-day-old colonies in TSB broth with NaCl concentrations of 0%, 3%, 6%, and 9% (w/v). Each bacterial strain was individually adjusted to an optical density at 600 nm (OD₆₀₀) of 0.1 before use. For the consortium, equal volumes of the two strains were mixed after OD₆₀₀ normalization, maintaining a final combined OD₆₀₀ of 0.1.

To measure indole-3-acetic acid (IAA) production, a colorimetric method based on Salkowski's technique (Bric et al. 1991) was employed. Cultures supplemented with 0.1% tryptophan were incubated at 25°C for 5 days. The supernatant was mixed in a 1:1 ratio with Salkowski reagent (0.5 M FeCl3: distilled water: concentrated H₂SO₄ = 1:50:30, v/v/v), kept in the dark for 30 min, and absorbance was measured at 530 nm.

Proline levels were assessed using a modified method by Bates et al. (1973). Cultures were incubated at 25°C for 3 days, after which the supernatant was treated with 3% sulfosalicylic acid and centrifuged. The supernatant was then combined with glacial acetic acid and acidic ninhydrin (1.25 g ninhydrin in 30 mL glacial acetic acid and 20 mL 6 M phosphoric acid) in a 1:1 ratio. The mixture was incubated at 90°C for 1 h, cooled, and extracted with toluene. Absorbance was measured at 520 nm.

Biofilm formation were measured using the Biofilm Formation Assay Kit (B601, Dojindo Laboratories) following the manufacture’s instruction.

For phosphate solubilization, the Murphy and Riley (1962) were applied. Cultures were grown in National Botanical Research Institute phosphate (NBRIP) broth for 7 days with NaCl concentrations (0%, 3%, 6%, and 9% (w/v)). The supernatant was combined with molybdate blue color reagent in a 1:1 ratio, and absorbance was measured at 882 nm using a KH₂PO₄ standard solution for phosphate quantification.

Exopolysaccharide (EPS) synthesis was assessed following Duckworth et al. (1999). Cultures were incubated at 25°C for 3 days, then treated with three volumes of chilled acetone and dried at 60°C overnight. EPS production was quantified by measuring the dry weight.

Siderophore production was evaluated using a modified method of Alexander and Zuberer (1991). Isolates were grown in MA broth with NaCl (0, 3%, 6%, and 9% w/v) at 25°C for 72 h. The supernatant was mixed in a 1:1 ratio with CAS reagent, left for 20 min, and absorbance was measured at 630 nm. Siderophore production was expressed as percent siderophore units (psu) using the formula: psu = (Ar-As) × 100/Ar, where Ar is the absorbance of the CAS solution with uninoculated broth, and As is the absorbance with supernatant.

Following the protocol by Tadayuki Iwase et al. (2013), isolates were incubated at 25°C for 3 days. The bacterial cell pellet was resuspended in physiological saline, mixed with 1% Triton X-100 and 30% hydrogen peroxide, and the height of oxygen bubble was measured to assess enzyme activity against a standard curve.

Quantitative amylase assays were conducted based on Xiao et al., with modifications (2006). Isolates were incubated on TSA medium containing 0.2% starch at 25°C for 48 h. The supernatant was stained with iodine reagent (5 mM I2 and 5 mM KI), and absorbance was measured at 580 nm. Amylase activity was quantified by calculating the residual starch concentration using a calibration curve based on the starch consumed over time.

**Supplementary table**

**Table S1. Primer list for qRT-PCR in this study.**

| **Name** | **Sequence 5‘-3’** |
| --- | --- |
| RD29A-qRT-F | CCTGAAGTGATCGATGCACCAG |
| RD29A-qRT-R | TGGTGTAATCGGAAGACACGAC |
| RD20-qRT-F | TTAGCTCCGGTCACCAGTCA |
| RD20-qRT-R | CATGTATGGTTTTGGTAATGTTTCC |
| KIN1-qRT-F | CCAACAAGAATGCCTTCCAAGC |
| KIN1-qRT-R | GCTGCCGCATCCGATACACT |
| TUBULIN2-qRT-F | TGGCATCAACTTTCATTGGA |
| TUBULIN2-qRT-R | ATGTTGCTCTCCGCTTCTGT |
| BS-16S-F | CAAGTACCGTTCGAATAGGGC |
| BS-16S-R | TGGCTCCTAAAAGGTTACCTC |
| tapA-F | TGCTTACAATTTTCCGATGATACAAG |
| tapA-R | ATCTGATATGTGCAAATCACTTTGATC |
| epsA-F | GCTGCGAAATATGGTCATGG |
| epsA-R | ACATCAGGAACGGAGCCTAA |

**Table S2. Summary of two-way ANOVA showing the effects of salt stress, bacterial treatment, and their interaction on physiological traits in**

***Arabidopsis* and cabbage.** Significance levels: ns, not significant (P ≥ 0.05); * P < 0.05; ** P < 0.01; *** P < 0.001; **** P < 0.0001.

| **Species** | **Trait** | **Source of Variation** | | | | | | | | |
| --- | --- | --- | --- | --- | --- | --- | --- | --- | --- | --- |
|  |  | **Interaction** | | | **Salt stress** | | | **Bacterial treatment** | | |
|  |  | **variation** | **P value** | **Significance** | **variation** | **P value** | **Significance** | **variation** | **P value** | **Significance** |
| *Arabidopsis* | Rosette size | 9.59% | <0.0001 | **** | 47.30% | <0.0001 | **** | 40.14% | <0.0001 | **** |
|  | Shoot fresh weight | 10.87% | <0.0001 | **** | 46.15% | <0.0001 | **** | 40.58% | <0.0001 | **** |
|  | Root fresh weight | 8.52% | <0.0001 | **** | 32.97% | <0.0001 | **** | 56.10% | <0.0001 | **** |
|  | Chlorophyll | 19.46% | <0.0001 | **** | 25.58% | <0.0001 | **** | 32.73% | <0.0001 | **** |
|  | CoroNa Green | 19.61% | <0.0001 | **** | 53.30% | <0.0001 | **** | 17.45% | <0.0001 | **** |
|  | K^+^contents | 1.40% | 0.0003 | *** | 96.10% | <0.0001 | **** | 0.11% | 0.6 | ns |
|  | Na^+^contents | 0.22% | 0.0838 | ns | 98.16% | <0.0001 | **** | 0.41% | 0.0084 | ** |
|  | K^+^/Na^+^ratio | 0.34% | 0.0911 | ns | 97.31% | <0.0001 | **** | 0.41% | 0.0508 | ns |
|  | DCF fluorescence | 30.07% | <0.0001 | **** | 34.82% | <0.0001 | **** | 24.21% | <0.0001 | **** |
|  | MDA content | 6.16% | <0.0001 | **** | 76.78% | <0.0001 | **** | 8.29% | <0.0001 | **** |
|  | POD activity | 7.16% | <0.0001 | **** | 84.92% | <0.0001 | **** | 4.99% | <0.0001 | **** |
|  | SOD activity | 13.15% | <0.0001 | **** | 54.55% | <0.0001 | **** | 31.12% | <0.0001 | **** |
| Cabbage | Shoot fresh weight | 0.59% | 0.0362 | * | 55.60% | <0.0001 | **** | 33.86% | <0.0001 | **** |
|  | Shoot dry weight | 0.28% | 0.5876 | ns | 56.75% | <0.0001 | **** | 30.33% | <0.0001 | **** |
|  | Shoot water content | 3.71% | 0.1118 | ns | 21.26% | <0.0001 | **** | 22.13% | <0.0001 | **** |
|  | Root fresh weight | 0.59% | 0.0192 | * | 73.51% | <0.0001 | **** | 20.89% | <0.0001 | **** |
|  | Root dry weight | 0.52% | 0.6985 | ns | 50.30% | <0.0001 | **** | 17.40% | <0.0001 | **** |
|  | Root water content | 0.17% | 0.9847 | ns | 1.20% | 0.3023 | ns | 0.18% | 0.9832 | ns |
|  | Chlorophyll | 14.90% | <0.0001 | **** | 14.21% | <0.0001 | **** | 33.45% | <0.0001 | **** |
|  | Na^+^ | 0.81% | <0.0001 | **** | 97.78% | <0.0001 | **** | 1.29% | <0.0001 | **** |
|  | K+ | 2.17% | 0.0005 | *** | 91.81% | <0.0001 | **** | 4.02% | 0.0011 | ** |
|  | K^+^/Na^+^ ratio | 1.01% | <0.0001 | **** | 96.79% | <0.0001 | **** | 1.57% | <0.0001 | **** |
|  | POD | 4.46% | 0.0909 | ns | 76.66% | <0.0001 | **** | 4.16% | 0.1074 | ns |
|  | SOD | 7.97% | <0.0001 | **** | 70.88% | <0.0001 | **** | 16.45% | <0.0001 | **** |
|  | MDA | 5.31% | 0.0011 | ** | 82.85% | <0.0001 | **** | 6.11% | 0.0005 | *** |
|  | Proline | 0.81% | 0.0283 | * | 88.27% | <0.0001 | **** | 6.07% | 0.0002 | *** |

**Table S3. Effects of *Kushneria* treatment on ion content, antioxidant enzyme activities, proline, and MDA in plants under salt-stressed and non- stressed conditions.** Different letters indicate statistically significant differences between bacteria-treated and untreated groups, based on two-way ANOVA (p < 0.05).

| **Condition Treatment** | **Na⁺ (mg/g DW)** | **K⁺ (mg/g DW)** | **K⁺/Na⁺** | **POD**  **(U/mg pro)** | **SOD**  **(U/mg pro)** | **Proline (µmol/g FW)** | **MDA**  **(nmol/g FW)** |
| --- | --- | --- | --- | --- | --- | --- | --- |
| Non-stressed CK | 2.90 ± 0.04 ^a^ | 44.20 ± 1.80 ^a^ | 15.27 ± 0.79 ^b^ | 32.20 ± 4.06 ^a^ | 329.75 ± 15.98 ^a^ | 1.58 ± 0.08 ^a^ | 12.62 ± 1.44 ^a^ |
| Kk | 2.48 ± 0.02 ^b^ | 42.43 ± 1.08 ^a^ | 17.10 ± 0.48 ^b^ | 33.80 ± 4.96 ^a^ | 367.75 ± 5.17 ^a^ | 1.82 ± 0.09 ^a^ | 13.30 ± 1.21 ^a^ |
| Km | 2.48 ± 0.02 ^b^ | 46.20 ± 0.60 ^a^ | 18.68 ± 0.37 ^a^ | 36.80 ± 5.80 ^a^ | 366.25 ± 5.60 ^a^ | 1.84 ± 0.09 ^a^ | 11.54 ± 1.30 ^a^ |
| Kkm | 2.22 ± 0.03 ^c^ | 45.40 ± 0.51 ^a^ | 20.47 ± 0.48 ^a^ | 33.60 ± 3.15 ^a^ | 368.00 ± 6.48 ^a^ | 1.82 ± 0.07 ^a^ | 11.86 ± 1.76 ^a^ |
| Salt- stressed CK-SA | 26.24 ± 0.40 ^a^ | 23.08 ± 0.29 ^b^ | 0.88 ± 0.012 ^c^ | 54.20 ± 2.62 ^b^ | 427.50 ± 18.70 ^c^ | 2.56 ± 0.08 ^c^ | 41.35 ± 2.97 ^a^ |
| Kk-SA | 22.65 ± 0.31 ^b^ | 22.41 ± 0.26 ^b^ | 0.99 ± 0.02 ^b^ | 63.80 ± 0.89 ^a^ | 591.50 ± 13.17 ^b^ | 2.93 ± 0.05 ^b^ | 31.26 ± 0.83 ^b^ |
| Km-SA | 22.07 ± 0.31 ^b^ | 23.15 ± 0.42 ^b^ | 1.05 ± 0.02 ^b^ | 57.20 ± 0.77 ^b^ | 602.00 ± 32.03 ^b^ | 2.89 ± 0.01 ^b^ | 32.66 ± 1.06 ^b^ |
| Kkm-SA | 20.53 ± 0.05 ^c^ | 30.56 ± 0.55 ^a^ | 1.49 ± 0.03 ^a^ | 70.40 ± 1.63 ^a^ | 661.00 ± 7.43 ^a^ | 3.09 ± 0.10 ^a^ | 26.50 ± 0.82 ^c^ |

2.0


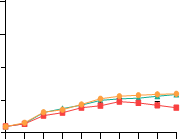

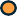

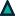


Kk
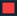
Km Kkm

**0% Na CI**

2.0

Kk
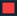
Km

Kkm

**3% Na CI**

1.5 1.5

1.0 1.0

OD600

OD600

0.5 0.5

0

0 8 16 24 32 40 48 56 64 72h

0

0 8 16 24 32 40 48 56 64 72h

2.0


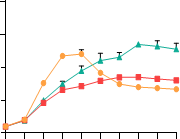

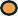

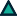

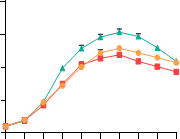

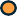

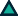


Kk Km Kkm

**6% Na CI**


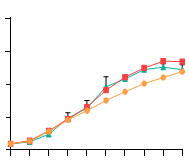

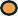

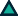


2.0

Kk Km Kkm

**9% Na CI**

1.5

1.0

0.5

0

0 8 16 24 32 40 48 56 64 72h

1.5

1.0

OD600

OD600

0.5

0

0 8 16 24 32 40 48 56 64 72h

**Figure S1. Growth curves of *Kk*, *Km*, and *Kkm* strains under different NaCl concentrations.** Bacterial cultures were incubated in media containing 0%, 3%, 6%, or 9% NaCl, and growth was monitored by measuring optical density at 600 nm (OD600) over a 72-hour period. Each point represents the mean ± standard deviation of three biological replicates.


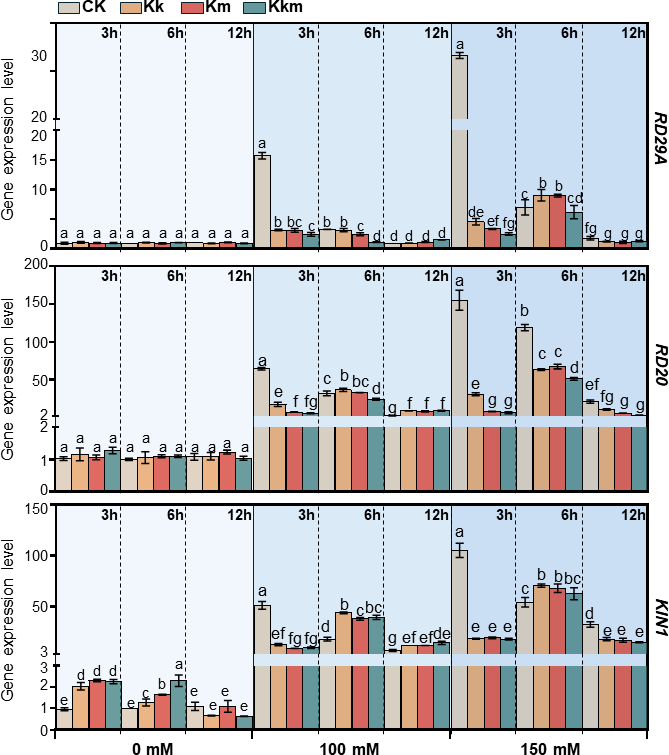


**Figure S2. Expression levels of salt stress–responsive genes in *Arabidopsis* seedlings following *Kushneria* inoculation under different salt concentrations.** Relative expressions of *RD29A*, *RD20*, and *KIN1* in *Arabidopsis* shoots treated with Kk, Km, and Kkm under 0, 100, and 150 mM NaCl conditions at 3 h, 6 h, and 12 h post-treatment. Expression levels were normalized to the endogenous control gene *TUBULIN2* (*TUB2*), and values are presented relative to the untreated control (CK). Bars represent mean ± SD of three independent biological replicates. A broken Y-axis was applied to allow comparison across genes with different expression magnitudes.


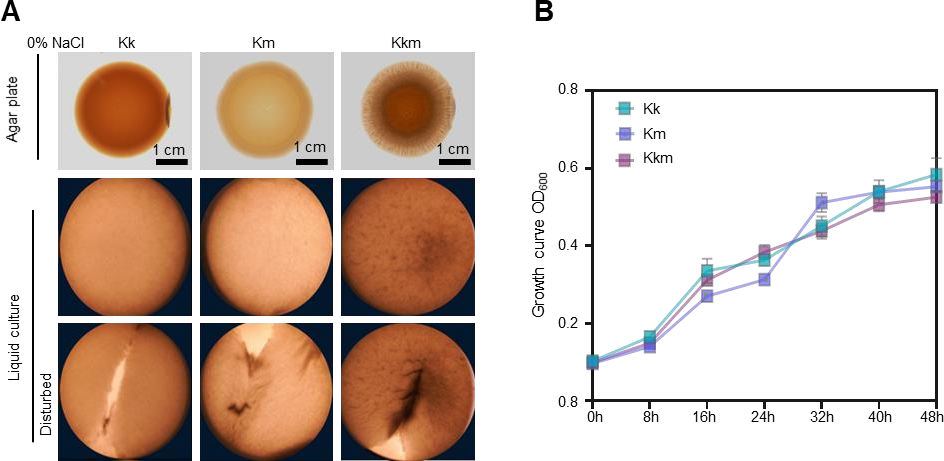


**Figure S3. Biofilm formation and growth dynamics of *Kushneria* spp.** (A) Biofilm formation by *Kushneria* spp. in solid and liquid media. The top row displays biofilms formed on plates, the middle row shows biofilms in liquid culture, and the bottom row presents disturbed liquid cultures. (B) Growth curve of *Kushneria* spp. under different treatments, measured by OD600 over a 48-hour period.


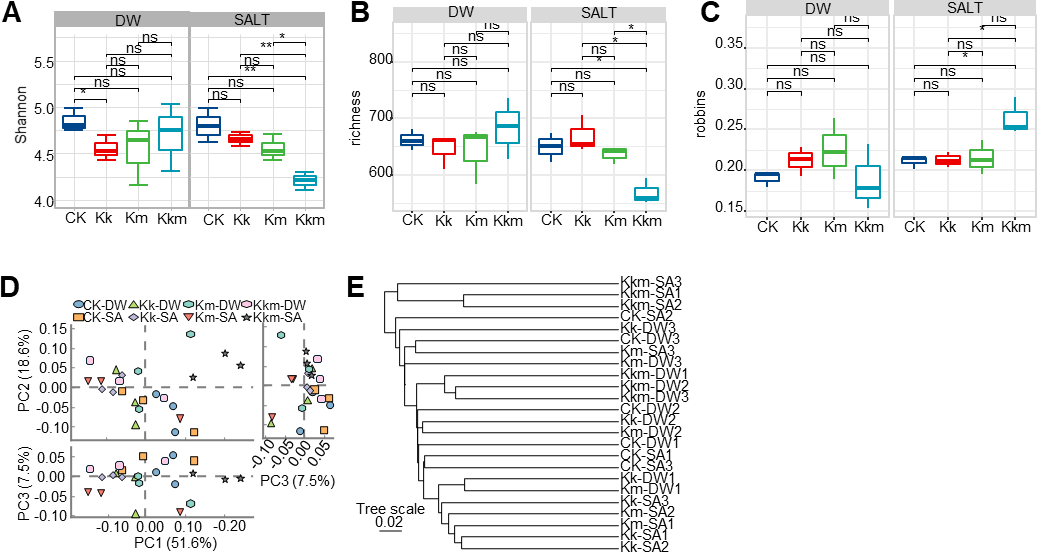


**Figure S4. Microbial community diversity and composition across treatments under non-saline (DW) and saline (SA) conditions.** (A) Shannon diversity index showing significant differences in microbial diversity among treatments (CK, Kk, Km, and Kkm) under DW and SA conditions.(B) Richness analysis comparing microbial diversity across treatments. (C) Pielou’s evenness index (Robbins) assessing community evenness. (D) Principal Component Analysis (PCA) of beta-diversity, illustrating variations in microbial composition. (E) Phylogenetic tree depicting microbial community clustering across samples, with a branch scale of 0.02. Significance levels: *p < 0.05, **p < 0.01, ns = not significant*.


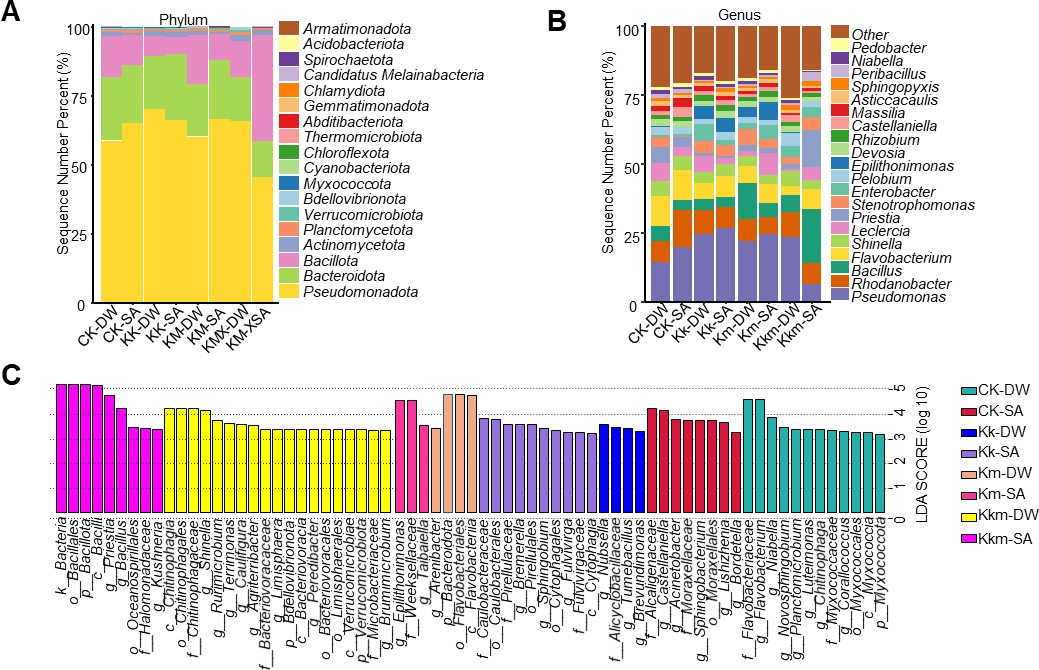


**Figure S5. Comparative analysis of microbial community composition and differentially enriched taxa across treatments. (A)** Relative abundance of microbial communities at the phylum level. **(B)** Relative abundance of microbial communities at the genus level. **(C)** LEfSe analysis highlighting significantly enriched genera across different treatments (CK, Kk, Km, and Kkm) under both DW and SA conditions.


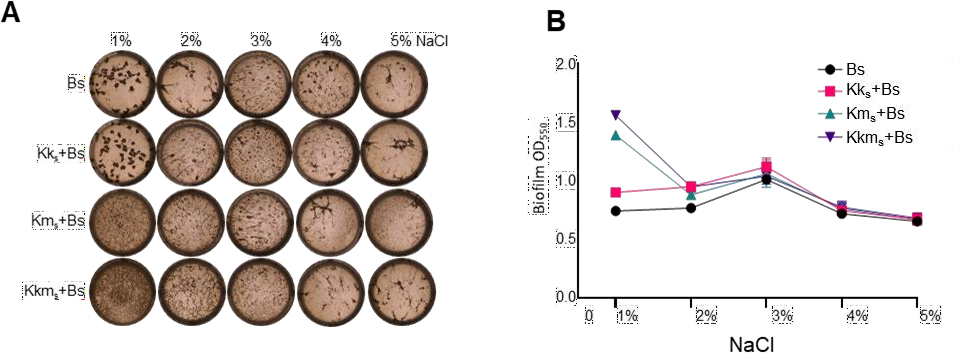


**Figure S6. Effect of *Kushneria* supernatant on *Bacillus subtilis* biofilm formation under different NaCl concentrations. (A)** Representative images depicting biofilm formation by *B. subtilis* alone (Bs) and in co-culture with *K. konosiri* supernatant (KkS + Bs), *K. marisflavi* supernatant (KmS + Bs), and the combined supernatant (Kkms + Bs) under 1%, 2%, 3%, 4%, and 5% NaCl conditions. **(B)** Quantitative analysis of biofilm biomass (OD550) across different treatments, demonstrating the influence of *Kushneria* supernatants on *B. subtilis* biofilm formation at varying salt concentrations.

# References

Alexander, D., D. Zuberer (1991) Use of chrome azurol S reagents to evaluate siderophore production by rhizosphere bacteria. Biology and Fertility of soils 12:39-45. https://doi.org/10.1007/BF00369386

Bates L.S., R.P. Waldren, I.D. Teare (1973) Rapid determination of free proline for water-stress studies. Plant and soil 39:205-207. https://doi.org/10.1007/BF00018060

Bric, J.M., R.M. Bostock, S.E. Silverstone (1991) Rapid in situ assay for indoleacetic Acid production by bacteria immobilized on a nitrocellulose membrane. Applied and Environmental Microbiology 57:535-538. https://doi.org/10.1128/aem.57.2.535-538.1991

Duckworth, A., W. Grant, A. Gambacorta (1999) *Haloarcula* spp. able to bio-synthesize exo endopolymers. *Journal of Industrial Microbiology and Biotechnology* 23: 489496. https://doi.org/10.1038/ sj.jim.2900738.

Iwase, T., A. Tajima, S. Sugimoto, K. Okuda, I. Hironaka, Y. Kamata, K. Takada, Y. Mizunoe (2013) A simple assay for measuring catalase activity: a visual approach. Scientific Reports 3:3081. https://doi.org/10.1038/srep03081.

Murphy, J., J.P. Riley (1962) A modified single solution method for the determination of phosphate in natural waters. Analytica chimica acta 27:31-36. https://doi.org/10.1016/S0003-2670(00)88444-5.

Xiao, Z., R. Storms, A. Tsang (2006) A quantitative starch-iodine method for measuring alpha-amylase and glucoamylase activities. Analytical Biochemistry 351:146-148. https://doi.org/10.1016/ j.ab.2006.01.036.
